# Efficacy and safety of hypoglycemic agents on gestational diabetes mellitus in women: A Bayesian network analysis of randomized controlled trials

**DOI:** 10.3389/fpubh.2022.980578

**Published:** 2022-12-02

**Authors:** Ting Wang, Yingyu Jing, Haonan Guo, Jing Xu, Man Wang, Lili Huang, Huan Chen, Wei Cui, Lin Song, Xiang Liu, Bo Sun, Ning Wang

**Affiliations:** ^1^Department of Endocrinology, The Second Affiliated Hospital of Xi'an Jiaotong University, Xi'an, China; ^2^Department of Respiratory Medicine, Xi'an People's Hospital (Xi'an No. 4 Hospital), Xi'an, China; ^3^Department of Endocrinology and Second Department of Geriatrics, The First Affiliated Hospital of Xi'an Jiaotong University, Xi'an, China; ^4^Department of Cardiovascular Medicine, Xi'an People's Hospital (Xi'an No. 4 Hospital), Xi'an, China; ^5^Department of Medical Ultrasound, The Second Affiliated Hospital of Xi'an Jiaotong University, Xi'an, China; ^6^Department of Physiology and Pathophysiology, School of Basic Medical Sciences, Xi'an Jiaotong University Health Science Center, Xi'an, China; ^7^Chinese Journal of Woman and Child Health Research, Xi'an, China; ^8^Postdoctoral Research Station, School of Nursing, Health Science Center, Xi'an Jiaotong University, Xi'an, China

**Keywords:** gestational diabetes mellitus, metformin, glyburide, insulin, Bayesian network analysis, randomized controlled trials

## Abstract

**Objective:**

To compare the efficacy and safety of metformin, glyburide, and insulin for GDM, we conducted a subgroup analysis of outcomes for women with GDM according to the International Association of Diabetes and Pregnancy Study Groups (IADPSG) diagnostic criteria.

**Methods:**

We searched the NCBI, Embase, and Web of Science databases from inception to March 2022. Randomized controlled trials (RCTs) that compared the outcomes of hypoglycemic agents in women with GDM were included. Bayesian network analysis was employed.

**Results:**

A total of 29 RCTs were included. Metformin was estimated to lead to a slight improvement in total gestational weight gain (WMD – 1.24 kg, 95% CI −2.38, −0.09), a risk of unmet treatment target in the sensitivity analysis (OR 34.50, 95% CI 1.18–791.37) than insulin. The estimated effect of metformin showed improvements in birth weight than insulin (WMD – 102.58 g, 95% CI −180.45 to −25.49) and glyburide (WMD – 137.84 g, 95% CI −255.31 to −25.45), for hypoglycemia within 1 h of birth than insulin (OR 0.65, 95% CI 0.47 to 0.84). The improvement in the estimated effect of metformin for hypoglycemia within 1 h of birth still existed when compared with glyburide (OR 0.41, 95% CI 0.26 to 0.66), whether in the IADPSG group (OR 0.33, 95% CI 0.12 to 0.92) or not (OR 0.43, 95% CI 0.20 to 0.98).

**Conclusion:**

Metformin is beneficial for GDM women to control total GWG compared with insulin, regulate fetal birth weight more than insulin and glyburide, and increase the risk of unmet treatment targets compared with insulin. Compared to metformin, glyburide is associated with neonatal hypoglycemia.

## Introduction

Gestational diabetes mellitus (GDM) is a common complication among pregnant women that usually occurs during the second and third trimesters (1). However, due to differences in GDM diagnostic criteria around the world, the reported prevalence of GDM also varies (2, 3). Due to poorly controlled blood glucose, GDM increases the risk of adverse gestational complications for both the mother and fetus (4, 5). However, with the recent deepening of our understanding of GDM, the use of stricter criteria for the diagnosis of GDM (IADPSG criteria) is recommended, in turn facilitating the management of blood glucose during pregnancy (1). Following a diagnosis of GDM, women are advised to control their blood glucose levels with diet and exercise. However, for those who fail to control their blood glucose, hypoglycemic drugs should be used including insulin (6–8), metformin (6, 8), glyburide (7), and in some studies, acarbose (9). Insulin is typically recommended as the first-line hypoglycemic therapy for women with GDM as it has a limited ability to cross the placental barrier (10). However, improper administration of insulin injections may increase the risk of hypoglycemia in pregnant women. Meanwhile, metformin and glyburide are secondary therapies for GDM, since they are able to enter fetal circulation through the placental barrier (11) although there are no reports of fetal malformations. Metformin is an oral hypoglycemic agent that increases glucose uptake and utilization in skeletal muscle, insulin sensitivity, and the promotion of glycolysis (12). Glyburide is an insulin secretion-promoting agent adapted for GDM with dysfunction of insulin secretion or intolerance to insulin injections (13, 14).

On a database search, we found four published network meta-analyses (NMA) on the use of hypoglycemic agents, including randomized controlled trials (RCTs), and compared the results to those of our NMA (Supplementary Tables 1–4). Three studies (15–17) compared the effects of metformin, insulin, and glyburide, and Jiang et al. (17) compared metformin, insulin, glyburide, and acarbose; however, the number of studies on acarbose is limited. Jiang et al. (17) included macrosomia, a subtype of LGA. Bidhendi Yarandi et al. (16) combined the outcomes of fetal and maternal complications during pregnancy, and had an increased number of studies but lacked specific outcomes. Yu et al. (15) listed respiratory distress syndrome (RDS) as an outcome, which should be classified as “admission to the Neonatal Intensive Care Unit (NICU),” and therefore, “anomaly” is not a specific diagnosis. Musa et al. (18) used outcomes based on the core outcome and measurement set (COS) (19), which is objective and consistent with clinical practice; however, there was a lack of comparisons between the outcomes of metformin and glyburide treatments by network analysis and pairwise meta-analysis. More importantly, the inconsistencies among diagnostic criteria for GDM for the included RCTs (18) may lead to heterogeneity among studies.

In this network meta-analysis, we aimed to update the recently published high-quality RCTs to compare the effect of the three treatments on core outcomes (COS) and provide more evidence for clinicians to choose hypoglycemic agents for women with GDM. Furthermore, because of the existence of heterogeneity between studies and statistical rigor, the network meta-analysis has intrinsic limitations, and so we conducted sensitivity and subgroup analysis on some outcomes according to the IADPSG diagnostic criteria.

## Materials and methods

The data were analyzed following the PRISMA statement and extension statement, with PROSPERO registration number CRD42022304011.

### Search strategy

We searched for all pregnancy-usable dosage forms of insulin, including human insulin, insulin isophane, insulin aspart, insulin lispro, insulin detemir, and insulin glargine. We carried out an extensive search strategy on the National Center for Biotechnology Information (NCBI), Embase, and Web of Science databases, using subject, related entry terms in NeSH, and Emtree in Embase to ensure that all relevant publications were captured. In addition, we searched PubMed for published studies on similar topic meta-analyses, and each included RCTs was retrieved if any was missed. EndNote was used as reference management software. Our search strategy is shown in Supplementary Table 5.

### Eligibility criteria

Randomized controlled trials published in English until 10 March 2022 were included. RCT participants had a diagnosis of GDM; they were divided into groups and treated with different pharmacological interventions and the efficacy of each treatment was measured. Only metformin, glyburide, and some pregnancy-usable dosage forms of insulin were included in the Bayesian network analysis. Owing to the limited number of studies, studies that compared different insulin regimens (intensive insulin vs. standard insulin) and different insulin dosage forms were excluded. We also excluded some studies that compared treatments and placebo/medical nutrition therapy (MNT)/diet, as well as several multi-arm studies of uncommon oral treatments (acarbose or the combination of hypoglycemic agents). Abstracts, letters, editorials, and conference presentations were also excluded. The diagnostic criteria for GDM lack a unified standard in different regions, which may be one of the reasons for the observed heterogeneity among studies. Due to the small number of valid studies, only some outcomes were analyzed according to the different diagnostic criteria of GDM.

### Data collection and quality measurement

We used EndNote to screen titles and abstracts after removing duplicates, and WN and GHN independently completed the work. In the case of disagreement between the two, the studies in question were discussed and an agreement was reached together with a third investigator (JYY). The full texts of the screened studies were evaluated according to the criteria of WN and GHN, and disagreements were resolved unanimously by the third investigator (JYY). Then, the data were extracted and checked by WN, GHN, JYY, HLL, and CH. Variables included author name, publication year, country, setting, period, criteria, design, participants, groups, GDM diagnostic criteria, pre-intervention, target blood glucose, sample sizes of the primary and secondary outcomes of the interventions, and the number of events in the studied arms (Supplementary Table 6). The quality of these studies was independently assessed using the MethodologicAl STandards for Epidemiological Research (MASTER) scales by SL and CW (Supplementary Table 7). The MASTER scale comprises 36 safeguards categorized into seven methodological standards.

### Outcomes

The included studies reported a wide range of GDM outcomes. However, the core outcomes (COS) were adopted according to the Gestational Metabolic Group of the Qatar Metabolic Institute (19). The COS included 11 significant clinical outcomes affected by GDM, which were classified into three aspects: maternal metabolic outcomes, fetal outcomes, and pregnancy outcomes. Maternal metabolic outcomes included total gestational weight gain (GWG), maternal hypoglycemia, mean fasting plasma glucose, mean postprandial glucose, and glycemic target unmet. Birth weight, large for gestational age (LGA), neonatal death, stillbirth, neonatal intensive care unit (NICU) admission, and hypoglycemia within 1 h of birth were included in the fetal outcomes. Pregnancy outcomes mainly include assisted labor, cesarean section, pre-term delivery, pregnancy induced hypertension, preeclampsia, and emergency cesarean section.

### Data analysis

Statistical analysis was performed using Review Manager software (version 5.3) and GeMTC GUI software (version 0.14.3). Pairwise meta-analyses were conducted using Review Manager software (version 5.3), and the pooled odds ratios (ORs) and their 95% confidence intervals (CIs) of dichotomies variables were calculated according to the number of outcome events in each RCT (Supplementary Tables 8–10). The weighted mean differences of the continuous variables were generated as effect sizes in the meta-analysis. This analytical framework was built on Bayesian network analysis to calculate the pooled ORs for dichotomous outcomes and WMDs for continuous outcomes using direct and indirect data for maternal metabolic, fetal, and pregnancy outcomes in women with GDM who were treated with metformin/glyburide/insulin. We run the results using consistent and inconsistent models. We used a random-effects model to minimize heterogeneity in the pairwise meta-analysis. Network maps were drawn using these data (Supplementary Figures 4–6).

We employed Cochran's *Q* and *H* indices to measure heterogeneity across pooled pairwise effects. The *H*-index was calculated as follows:


$$\begin{array}{l} H=\sqrt{\frac{\max\left[\max\left(1,n-1\right)\right],\;Q}{\max\left(1,n-1\right)}} \end{array}$$

“*n*” is the estimated number of studies, and *Q* is the Chi-squared from Cochran's *Q*. Sensitivity analyses were conducted by omitting a single study of the results of the weighted pooled index (*H*) > 3, which indicates the existence of heterogeneity (20). We accessed the publication bias by funnel plots. Subgroup analyses were conducted according to GDM diagnosis criteria; sensitivity analyses were employed if heterogeneity existed.

For the abnormal distributed variables which were reported as median and range or interquartile range, we used standard methods to convert them into the mean standard deviation (SD), and combined the mean and SD of variables as follows (21, 22):


$$\begin{array}{l} \bar{x}\;=\;\frac{\left({\bar{x}}_{1}\;\times \;n_{1}\;+\;\ldots {\bar{x}}_{n}\;\times \;n_{n}\right)}{n_{1}\;+\;\ldots +\;n_{n}}\\ \bar{SD}=\;\sqrt{\frac{\left[\left(\sqrt{SD_{1}}+\sqrt{{\bar{x}}_{1}}\right)\;\times \;n_{1}\;+\;\ldots +\left(\sqrt{SD_{n}}\;+\;\sqrt{{\bar{x}}_{n}}\right)\;\times \;n_{n}\right]}{\left(n_{1}+\ldots +n_{n}\right)}\;-\;\sqrt{\frac{\left({\bar{x}}_{1}\times n_{1}\;+\;\ldots +\;{\bar{x}}_{n}\;\times \;n_{n}\right)}{n_{1}\;+\;\ldots \;+\;n_{n}}}} \end{array}$$

## Results

### Identified studies

**Figure 1:**
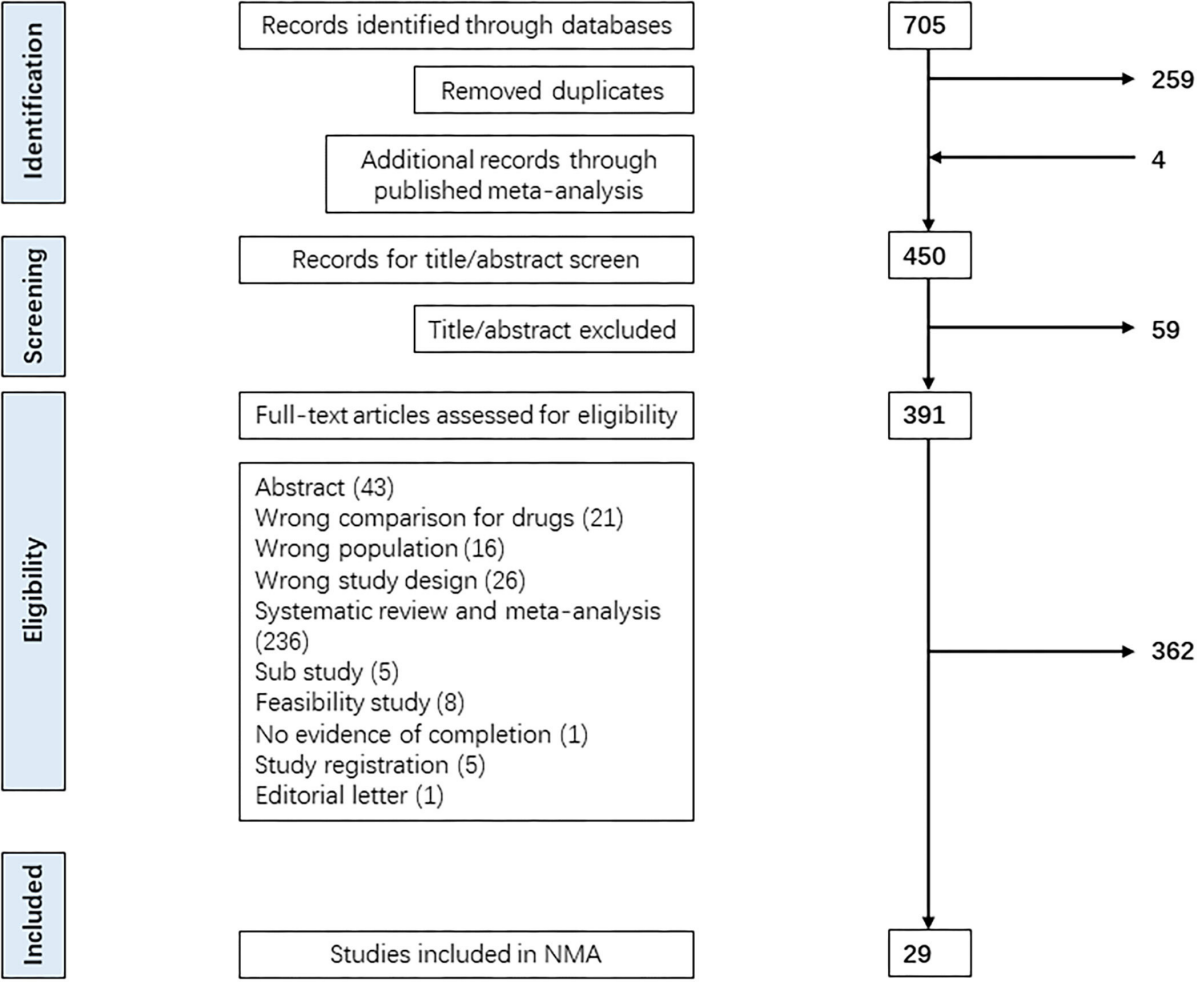
PRISMA flowchart for the selection of studies. Figure manifested the process of retrieving, evaluating, selecting, or excluding relevant studies from the database. We employed the preferred reporting items for systematic reviews and meta-analyses (PRISMA) diagram for the search.

The Bayesian network analysis included 29 RCTs. A total of 705 records were identified from MEDLINE, Embase, and Web of Science until the end of 16 January 2022, through a merged method of MeSH heading search strategy and the term: “diabetes, gestational” “randomized controlled trials” “hypoglycemic agents/insulin/insulin isophane/insulin aspart/insulin lispro/insulin determir/insulin glargine.” After removing duplicates and adding records through a published meta-analysis, we conducted a full-text search of 450 RCTs. A total of 421 RCTs were excluded for various reasons, as shown in the PRISMA flowchart (Figure 1). For trials which contained three arms, we dropped the medical nutrition therapy (MNT)/acarbose, and the other two arms were included. Finally, 29 RCTs fulfilled the inclusion criteria for the Bayesian network analysis.

### Baseline clinical characters

In the metformin-treated group (Table 1), the weighted mean clinical characters were 31.80 years (*n* = 19 arms) of age, 29.72 kg/m^2^ (*n* = 17 arms) for BMI, 2.6% of women with previous GDM (*n*= 4 arms), 28.28% primigravida women (*n* = 3 arms), with an OGTT of 5.61 mmol/L (*n*= 12 arms) for 0 h, and 9.72 mmol/L (*n* = 12 arms) for 2 h. For the insulin-treated group, the weighted mean clinical characters were 31.53 years (*n* = 24 arms) of age, 29.83 kg/m^2^ (*n* = 20 arms) for BMI, 20.2% women with previous GDM (*n* = 8 arms), 26.70% primigravida women (*n* = 3 arms), with an OGTT of 5.77 mmol/L (*n* = 15 arms) for 0 h, and 9.85 mmol/L (*n* = 15 arms) for 2 h. For the glyburide-treated group, the weighted mean clinical characters were 31.44 years (*n* = 11 arms) of age, 29.30 kg/m^2^ (*n* = 10 arms) for BMI, 30.2% women with previous GDM (*n* = 4 arms), 26.60% primigravida women (*n* = 2 arms), with an OGTT of 5.39 mmol/L (*n* = 5 arms) for 0 h, and 9.86 mmol/L (*n* = 4 arms) for 2 h. The diagnostic criteria for GDM for each RCT are listed in Supplementary Table 6.

**Table 1:** Baseline clinical characters of GDM women in different arms of the Bayesian network-analysis.

**Variable name**	**Treatment**	**Mean/No**.	**SD/%**	**Size**
**Age**				
	Metformin (19)	31.80	5.53	1,610
	Insulin (24)	31.53	5.59	2,503
	Glyburide (11)	31.44	5.96	1,428
**BMI**				
	Metformin (17)	29.72	6.12	1,542
	Insulin (20)	29.83	6.08	2,140
	Glyburide (10)	29.30	7.13	1,379
**Previous GDM**				
	Metformin (4)	183	2.6	686
	Insulin (8)	293	20.2	1,449
	Glyburide (4)	332	30.2	1,101
**Primigravida**				
	Metformin (3)	56	28.28	198
	Insulin (3)	51	26.70	191
	Glyburide (2)	25	26.60	94
**OGTT 0 h**				
	Metformin (12)	5.61	0.98	1,216
	Insulin (15)	5.77	1.76	1,574
	Glyburide (5)	5.39	1.15	780
**OGTT 2 h**				
	Metformin (12)	9.72	2.10	1,216
	Insulin (15)	9.85	2.28	1,527
	Glyburide (4)	9.86	1.83	753

### Origin of studies

Thirteen RCTs came from Alisa (India, Pakistan, Iran, and Israel), six from Europe (Finland, Mexico, Spain, France, and Macedonia), four from Africa (Egypt), four from North America (USA), two from South America (Brazil), and one from Oceania (New Zealand and Australia; Supplementary Table 6).

### Interventions, outcomes, and participants

The interventions in this network included metformin, glyburide, and insulin. Women were administered metformin at doses of 500–2,500 mg per day and glyburide was used at a low dose ranging from 0.625 to 20 mg per day. Insulin was recommended during pregnancy (regular insulin, rapid-acting insulin, insulin isophane, insulin aspart, insulin lispro, insulin detergent, and insulin glargine), and intensive insulin therapy was classified as insulin treatment. Fifteen RCTs compared the outcomes of metformin and insulin treatments, 10 RCTs compared the outcomes of glyburide and insulin treatments, and four RCTs compared the outcomes of metformin and glyburide treatments. A total of 5,782 participants from 29 RCTs were included.

### Comparative efficacy results

#### Maternal metabolic outcomes

Insulin showed a higher estimated effect than glyburide in total GWG (WMD 4.89 kg, 95% CI 1.10 to 8.67, Table 2). However, in the sensitivity analysis of total GWG, the estimated effect of metformin showed a slight improvement compared to insulin (WMD −1.24 kg, 95% CI −2.38 −0.09). The estimated effects of metformin indicated an increased risk of unmet treatment targets compared to insulin in the sensitivity analysis with a limited number of studies (OR 34.50, 95% CI 1.18–791.37). Other comparisons regarding the estimated effects of metformin-insulin, glyburide-insulin, and metformin-glyburide showed no statistically significant differences.

**Table 2:** The Bayesian network-analysis for maternal metabolic outcomes.

**Maternal metabolic outcomes**	**Treatment**	**Type of effect**	**Effect**	**95% CI**
*Total GWG* *H* = 5.6, *N* = 12, *n* = 2,592	Met vs. Ins	WMD	−1.72	−3.70 to 0.25
	Met vs. Gly	WMD	3.17	−0.78 to 7.09
	Ins vs. Gly	WMD	4.89^*^	1.10 to 8.67
*Sensitivity analysis of total GWG* *H* = 3.0, *N* = 9, *n* = 1,964	Met vs. Ins	WMD	−1.24^*^	−2.38 to −0.09
	Met vs. Gly	WMD	−0.05	−2.74 to 2.71
	Ins vs. Gly	WMD	1.19	−1.49 to 3.99
*Maternal hypoglycemia* *H* = 1.7, *N* = 5, *n* = 1,299	Met vs. Ins	OR	0.76	0.09 to 6.56
	Met vs. Gly	OR	0.25	0.03 to 2.86
	Ins vs. Gly	OR	0.32	0.03 to 4.81
*Mean plasma glucose* *H* = 2.4, *N* = 19, *n* = 4,000	Met vs. Ins	WMD	−0.06	−0.18 to 0.05
	Met vs. Gly	WMD	0.10	−0.07 to 0.26
	Ins vs. Gly	WMD	0.15	−0.00 to 0.32
*Mean postprandial glucose* *H* = 5.7 *N* = 19, *n* = 3,973	Met vs. Ins	WMD	−0.01	−0.36 to 0.33
	Met vs. Gly	WMD	−0.02	−0.50 to 0.44
	Ins vs. Gly	WMD	−0.01	−0.44 to 0.41
*Sensitivity analysis of mean postprandial glucose* *H* = 2.2 *N* = 18, *n* = 3,687	Met vs. Ins	WMD	−0.16	−0.34 to 0.01
	Met vs. Gly	WMD	−0.09	−0.33 to 0.14
	Ins vs. Gly	WMD	0.07	−0.15 to 0.28
*Treatment target unmet* *H* = 3.4, *N* = 4, *n* = 663	Met vs. Ins	OR	9.25	0.34 to 483.43
	Met vs. Gly	OR	0.28	0.00 to 87.69
	Ins vs. Gly	OR	0.03	0.00 to 19.15
*Sensitivity analysis of treatment target unmet* *H* = 2.483, *N* = 3, *n* = 463	Met vs. Ins	OR	34.50^*^	1.18 to 791.37
	Met vs. Gly	OR	0.28	0.00, 63.38
	Ins vs. Gly	OR	0.01	0.00, 3.46

H, weighted pooled index: The minimum value H can take is 1, and if H <3, minimal inconsistency presents.

N, number of trials.

n, number of participants per arm.

a* Significant statistical differences.

#### Fetal outcomes

Metformin showed an improved estimated effect on birth weight compared to insulin (WMD to 102.58 g, 95% CI −180.45 to −25.49, Table 3) and glyburide (WMD – 137.84 g, 95% CI −255.31 to −25.45). Similar results were obtained in the subgroup analysis of non-IADPSG, and no statistical differences in birth weight were observed among the three groups in the IADPSG group. Regarding the hypoglycemia within 1 h of the birth outcome, the estimated effect of metformin indicated improvements compared to insulin (OR 0.65, 95% CI 0.47 to 0.84) and glyburide (OR 0.41, 95% CI 0.26 to 0.66), with the ranking of the treatments for the improvement of hypoglycemia being metformin, insulin, and glyburide. In the subgroup analyses of hypoglycemia within 1 h of birth, the estimated effect of metformin showed improvements compared to glyburide in the IADPSG group (OR 0.33, 95% CI 0.12 to 0.92) and the non-IADPSG group (OR 0.43, 95% CI 0.20 to 0.98). The comparisons between the estimated effects of metformin-insulin, glyburide-insulin, and metformin-glyburide on other fetal outcomes were not statistically different.

**Table 3:** The Bayesian network-analysis for fetal outcomes.

**Fetal outcomes**	**Treatment**	**Type of effect**	**Effect**	**95% CI**
*Birth weight* *H* = 2.5, *N* = 23, *n* = 4949	Met vs. Ins	WMD	−102.58^*^	−180.45 to −25.49
	Met vs. Gly	WMD	−137.84^*^	−255.31 to −25.45
	Ins vs. Gly	WMD	−35.16	−146.08 to 71.86
Subgroup analysis of birthweight (IADPSG criteria)	Met vs. Ins	WMD	−113.37	−348.74 to 148.04
	Met vs. Gly	WMD	−125.14	−657.57 to 409.78
	Ins vs. Gly	WMD	−12.99	−486.34 to 451.88
Subgroup analysis of birthweight (non-IADPSG criteria)	Met vs. Ins	WMD	−84.78^*^	−165.37 to −1.34
	Met vs. Gly	WMD	−125.64^*^	−247.54 to −16.74
	Ins vs. Gly	WMD	−41.16	−160.32 to 65.11
*LGA* *H* = 1.2, *N* = 15, *n* = 3,229	Met vs. Ins	OR	0.73	0.47 to 1.10
	Met vs. Gly	OR	0.63	0.27 to 1.17
	Ins vs. Gly	OR	0.86	0.40 to 1.48
*Neonatal death* *H* = 1.0, *N* = 10, *n* = 2,265	Met vs. Ins	OR	0.29	0.00 to 34.24
	Met vs. Gly	OR	0.30	0.00 to 1,454.94
	Ins vs. Gly	OR	1.21	0.00 to 1,377.31
*Still birth* *H* = not applicable, *N* = 4, *n* = 731	Met vs. Ins	OR	74.76	0.00 to 4,274.44
	Met vs. Gly	OR	24.61	0.00 to 1,502.44
	Ins vs. Gly	OR	0.41	0.01 to 6.98
*Hypoglycemia within 1 h of birth* *H* = 1.0, *N* = 24, *n* = 5,248	Met vs. Ins	OR	0.65^*^	0.47 to 0.84
	Met vs. Gly	OR	0.41^*^	0.26 to 0.66
	Ins vs. Gly	OR	0.63^*^	0.43 to 0.99
Subgroup analysis of hypoglycemia within 1 h of birth (IADPSG criteria)				
	Met vs. Ins	OR	0.59	0.31 to 1.09
	Met vs. Gly	OR	0.33^*^	0.12 to 0.92
	Ins vs. Gly	OR	0.55	0.25 to 1.25
Subgroup analysis of hypoglycemia within 1 h of birth (non-IADPSG criteria)				
	Met vs. Ins	OR	0.64	0.37 to 1.02
	Met vs. Gly	OR	0.43^*^	0.20 to 0.98
	Ins vs. Gly	OR	0.69	0.34 to 1.46
*NICU* *H* = 1.0, *N* = 19, *n* = 4,105	Met vs. Ins	OR	0.83	0.65 to 1.07
	Met vs. Gly	OR	0.97	0.63 to 1.51
	Ins vs. Gly	OR	1.17	0.80 to 1.74

H, weighted pooled index: The minimum value H can take is 1, and if H <3, minimal inconsistency presents.

N, number of trials.

n, number of participants per arm.

* Significant statistical differences.

#### Pregnancy outcomes

Metformin showed an improvement in the estimated effects of cesarean section (OR 0.82, 95% CI 0.63–1.11, Table 4), pregnancy induced hypertension (OR 0.58, 95% CI 0.34–1.12), and preeclampsia compared with insulin (OR 0.74, 95% CI 0.32–1.36); however, there is limited evidence against the hypothesis that metformin and insulin are equivalent on pregnancy outcomes. The estimated effects of the comparisons of metformin-insulin, glyburide-insulin, and metformin-glyburide on other fetal outcomes were not statistically different.

**Table 4:** The Bayesian network-analysis for pregnancy outcomes.

**Pregnancy outcomes**	**Treatment**	**Type of effect**	**Effect**	**95% CI**
*Assisted labor (non-cesarean)* *H* = 1.0, *N* = 8, *n* = 1,765	Met vs. Ins	OR	1.02	0.67 to 2.08
	Met vs. Gly	OR	1.04	0.38 to 2.67
	Ins vs. Gly	OR	0.87	0.38 to 1.97
*Cesarean* *H* = 1.4, *N* = 19, *n* = 3,714	Met vs. Ins	OR	0.82	0.63 to 1.11
	Met vs. Gly	OR	1.15	0.78 to 1.89
	Ins vs. Gly	OR	1.41	0.94 to 2.25
*Emergency cesarean* *H* = 1.6, *N* = 3, *n* = 1,106	Met vs. Ins	OR	1.32	0.64 to 2.86
	Met vs. Gly	OR	0.96	0.32 to 2.93
	Ins vs. Gly	OR	0.73	0.33 to 1.62
*Pregnancy induced hypertension* *H* = 1.2, *N* = 9, *n* = 2,311	Met vs. Ins	OR	0.58	0.34 to 1.12
	Met vs. Gly	OR	0.73	0.33 to 1.61
	Ins vs. Gly	OR	1.20	0.52 to 2.53
*Preeclampsia* *H* = 1.0, *N* = 7, *n* = 1,663	Met vs. Ins	OR	0.74	0.32 to 1.36
	Met vs. Gly	OR	0.51	0.03 to 5.32
	Ins vs. Gly	OR	0.70	0.05 to 8.45
*Pre-term delivery* *H* = 1.0, *N* = 8, *n* = 1,840	Met vs. Ins	OR	1.60	0.84 to 2.95
	Met vs. Gly	OR	1.20	0.51 to 3.09
	Ins vs. Gly	OR	0.76	0.34 to 1.91

H, weighted pooled index: The minimum value H can take is 1, and if H <3, minimal inconsistency is present.

N, number of trials.

n, number of participants per arm.

*Significant statistical differences.

#### Consistency and publication bias

Consistency across the network was measured using the average “H” statistic, which equaled 1 on the outcomes of neonatal death, hypoglycemia within 1 h of birth, NICU, assisted labor (non-cesarean), preeclampsia, pre-term delivery, thus indicating consistency for the effects above. Minimal inconsistencies were observed in maternal hypoglycemia, mean plasma glucose, birth weight, LGA, cesarean section, emergency cesarean section, and pregnancy-induced hypertension, as “H” <3. Mean postprandial glucose, treatment target unmet, and total GWG had an average “H” statistic ≥ 3, thus indicating significant inconsistency. While the first two improved after the sensitivity analyses there was hardly any improvement for total GWG.

#### Funnel plots

The comparison of the funnel plots of outcomes on maternal metabolic, fetal, and pregnancy showed no or mild dissymmetry for most of the outcomes above, except for the funnel plots on total GWG outcomes, mean plasma fasting glucose, mean postprandial glucose, and birth weight. The dissymmetry of the total GWG was probably due to the small number of included studies. Heterogeneity may also exist in the outcomes of mean plasma fasting glucose, mean postprandial glucose, and birth weight, which leads to some dissymmetry in their funnel plots (Supplementary Figures 1–3).

## Discussion

By combining direct and circumstantial evidence, we conducted an NMA on women with GDM to assess the effects of interventions with metformin, insulin, and glyburide. For the maternal metabolic outcomes, metformin treatment was correlated with a reduction in total GWG and an increased number of treatment targets unmet compared with the insulin group. In terms of fetal outcomes, metformin treatment resulted in lower birth weight compared to the insulin and glyburide groups, which is similar to the non-IADPSG criteria subgroup analysis; however, there were no significant differences in the subgroup analysis of the IADPSG criteria due to the limited number of studies. Meanwhile, glyburide is relevant to neonatal hypoglycemia within 1 h compared to the metformin group, regardless of whether the IADPSG criteria were employed. No significant differences were found in pregnancy outcomes.

Metformin had a reduced ability to increase total GWG compared to insulin. This is likely because insulin promotes the uptake of glucose by adipose tissue, which stimulates the re-esterification of free fatty acids into triglycerides in adipocytes (23), in turn leading to an increase in total GWG. However, the mechanism by which metformin improves blood glucose is through increasing the uptake of glucose by tissues, its utilization by skeletal muscle, insulin sensitivity, promotion of glycolysis, and inhibition of hepatic gluconeogenesis, which eventually leads to weight loss (12). Excessive GWG has many adverse effects on both the mother and offspring (24), and weight gain from the beginning of pregnancy to the present should be an important basis for selecting hypoglycemic agents.

Meanwhile, the increased number of pregnant women who did not meet treatment targets was associated with the use of metformin. Metformin is regarded as an insulin-sensitizing agent that is used as an oral hypoglycemic agent with limited power to control blood glucose levels during clinical practice (25). We have shown that there are three subtypes of GDM according to the heterogeneity in the physiological and pathological processes leading to hyperglycemia, which show different characteristics, risk factors, and insulin sensitivity alteration patterns (26, 27). In the GDM-resistant subtype, metformin may improve blood glucose levels. Indeed, the use of metformin has been shown to result in a long-term and more stable hypoglycemic effect than insulin/glyburide in women with resistant GDM, which may be associated with obesity-induced insulin resistance (28). GDM dysfunction and GDM-mixed subtypes are characterized by insufficient insulin secretion; therefore, the use of metformin may have limited effects. However, insulin can achieve tight maternal glucose control regardless of the subtype.

Metformin does not cause neonatal hypoglycemia and overgrowth. This is because, although it crosses the placental barrier, it acts by improving insulin sensitivity in fetal peripheral tissue rather than by promoting insulin secretion (29, 30), without leading to the accumulation of subcutaneous fat. Glyburide treatment in women with GDM is associated with neonatal hypoglycemia and overgrowth compared to metformin treatment, which is consistent with many published studies (17, 18, 28, 31, 32). Glyburide enters the fetus through the placenta and leads to excessive fetal insulin secretion (14). Then, once the fetus is born without the nutritional supply from the mother, high peripheral insulin levels can lead to neonatal hypoglycemia in the absence of glucose supplementation (33). Furthermore, a large proportion of women with GDM under glyburide treatment suffer from insulin secretion deficiency, due to elevated hormones in late pregnancy which aggravate glucose and lipid metabolism disorders (34), which may lead to the enhancement of fetal circumstance blood glucose and insulin level.

The recommendations for metformin use in women vary according to different associations. China has a high prevalence of GDM, but yet there are limited published RCTs on oral hypoglycemic agents for GDM, which may be due to the restriction of RCTs' ethical review, and the recommendation of the current guidelines. The Professional Committee of Gestational Diabetes of the China Maternal and Child Health Association recommended insulin as the first-line hypoglycemic therapy for women with GDM based on the recommendations of the American Diabetes Association (35). However, for women with GDM who are intolerant or refuse to use insulin, such as those with poor compliance with insulin injections or inability to afford the cost of insulin, metformin could be used as an alternative for women with GDM without contraindications (35). Meanwhile, for pregnant women with T2DM, metformin could be used in cases in which diet and exercise cannot control blood glucose to the target range, or in those with significant insulin resistance with increased insulin dose but limited effect on blood glucose control (35). Similarly, the American College of Obstetricians and Gynecologists (ACOG) and American Diabetes Association (ADA) recommend insulin as the first-line therapy for women with GDM, with metformin and glyburide only used under the subjective or objective conditions listed above (36, 37). Metformin is not recommended for women with hypertension, preeclampsia, placental insufficiency, fetal growth restriction, and acidosis (37). However, according to the guidelines of the Society for Maternal-Fetal Medicine and the National Institute for Clinical Excellence (NICE), metformin is recommended for women with GDM after 20 weeks of gestation who cannot maintain blood glucose in the target range through diet and exercise (38). The restriction on the use of oral hypoglycemic drugs for women with GDM in different regions leads to limitations in large-scale worldwide RCTs, comparing the outcomes of using hypoglycemic drugs in women with GDM.

The advantages of this study are that we conducted an updated and comprehensive literature search and selected the outcomes based on the COS, with a biased assessment of each study using the MASTER scale. Owing to the limited number of studies, we performed subgroup analyses on parts of the outcomes according to the IADPSG diagnostic criteria. We also list the results of the Bayesian network analysis and pairwise meta-analysis.

## Limitation

The limitation of the studies includes that most of the participants in the studies came from urban, which may lead to selection bias. Due to the influence of the route of administration, unrealizable double blindness, different geographical area, and diagnostic criteria, heterogeneity could not be eliminated by sensitivity analyses, as we could not exclude the influence of age, BMI, region, ethnicity, target blood glucose control, and branded medicines. Although subgroup analyses of IADPSG and non-IADPSG were conducted, due to the limited number of studies, some outcomes have been heterogeneous. Next, we will continue to pay attention to such RCTs, and conduct further subgroup analyses of different GDM diagnoses. Finally, in combination with our previous studies (26, 27, 39), GDM subtypes should be considered as an important confounding factor that could affect the clinical efficacy of these oral hypoglycemic agents.

## Conclusion

In this Bayesian network analysis on hypoglycemic agents for women with GDM, metformin is beneficial to control total GWG and fetal birth weight, and glyburide is associated with neonatal hypoglycemia. Further RCTs on women with GDM should be conducted based on the widely accepted IADPSG diagnostic criteria. For women with GDM who have insulin resistance and no contraindications to metformin, the benefits of using metformin are not limited to controlling blood glucose stable, but also avoiding excessive weight gain for both mother and fetus during pregnancy, which is helpful to guide the use of hypoglycemic agents for GDM women.

## Data availability statement

The original contributions presented in the study are included in the article/Supplementary material, further inquiries can be directed to the corresponding authors.

## Author contributions

TW, NW, and BS designed the work presented in the article. TW, YJ, and HG completed the analysis and drafted and revised the article. JX, MW, LH, HC, WC, LS, and XL revised the article for critically important content. BS approved the final version to be published. All authors contributed to the article and approved the submitted version.

## Funding

We acknowledge grant funding of the Natural Science Foundation of Shaanxi Province (No. 2020GXLHY-029).

## Conflict of interest

The authors declare that the research was conducted in the absence of any commercial or financial relationships that could be construed as a potential conflict of interest.

## Publisher's note

All claims expressed in this article are solely those of the authors and do not necessarily represent those of their affiliated organizations, or those of the publisher, the editors and the reviewers. Any product that may be evaluated in this article, or claim that may be made by its manufacturer, is not guaranteed or endorsed by the publisher.
