# Supplementary material for: Efficacy and safety of hypoglycemic agents on gestational diabetes mellitus in women: A Bayesian network analysis of randomized controlled trials
Source: Front Public Health. 2022 Dec 2;10:980578. doi: 10.3389/fpubh.2022.980578 (PMC9757606; doi:10.3389/fpubh.2022.980578)
Supplement: Supplementary file 1 [file Data_Sheet_1.docx]

Supplementary Material

## Supplementary Tables

**Supplementary Table 1.** Our NMA results in comparison to Jiang 2015(Jiang et al., 2015)

| **No.** | **Outcome** | **Treatment favor** | **Consistency** |
| --- | --- | --- | --- |
| 1 | HbA1c | Not analyzed | Irrelevant outcome |
| 2 | Fasting glycemic | Not analyzed | Irrelevant outcome |
| 3 | Weight gain | Favors insulin | Consistent |
| 4 | Cesarean delivery | No difference | Consistent |
| 5 | Pre-eclampsia | No difference | Consistent |
| 6 | Birth weight | Favors glyburide | Consistent |
| 7 | Hypoglycemia | Favors glyburide | Consistent |
| 8 | Gestational age | Not analyzed | Irrelevant outcome |
| 9 | Premature birth | Favors metformin | Inconsistent |
| 10 | Macrosomia | Not analyzed | Irrelevant outcome |
| 11 | NICU | No difference | Consistent |

**Supplementary Table 2.** Our NMA results in comparison to Musa 2021(Musa et al., 2021)

| **No.** | **Outcome** | **Treatment favor** | | **Consistency** |
| --- | --- | --- | --- | --- |
| 1 | Gestational GWG | Favors insulin | | Consistent |
| 2 | Maternal hypoglycemia | Favors insulin | | Inconsistent |
| 3 | Mean plasma glucose | No difference | | Consistent |
| 4 | Mean postprandial | No difference | | Consistent |
| 5 | Treatment target unmet | No difference | | Consistent |
| 6 | Birth weight | Favors insulin | | Consistent |
| 7 | Large for gestational age | Favors insulin | | inconsistent |
| 8 | Neonatal death | No difference | | Consistent |
| 9 | Still Birth | No difference | | Consistent |
| 10 | Hypoglycemia within 1 h of birth | No difference | | inconsistent |
| 11 | NICU admission | No difference | | Consistent |
| 12 | Assisted labor (non-cesarean) | No difference | | Consistent |
| 13 | Cesarean section | No difference | | Consistent |
| 14 | Emergency cesarean section | No difference | | Consistent |
| 15 | Pregnancy induced hypertension | | No difference | Consistent |
| 16 | Preeclampsia | No difference | | Consistent |
| 17 | Pre-term delivery | No difference | | Consistent |

**Supplementary T****able 3.** Our NMA results in comparison to Yarandi 2021

| **No.** | **Outcome** | **Treatment favor** | **Consistency** |
| --- | --- | --- | --- |
| 1 | Risk of Birth Defect | Not analyzed | Irrelevant outcome |
| 2 | Risk of Birth Trauma | Not analyzed | Irrelevant outcome |
| 3 | Risk of Neonatal metabolic disturbance | Favors insulin | Inconsistent |
| 4 | Risk of Neonatal/Perinatal Mortality | No difference | Consistent |
| 5 | Risk of Complicated Pregnancy | Favors insulin | Inconsistent |
| 6 | Risk of Abnormal Delivery | Favors glyburide | Inconsistent |
| 7 | Risk of Abnormal Fetal Growth | Favors glyburide | Inconsistent |
| 8 | Risk of NICU Admission | Favors insulin and glyburide | inconsistent |
| 9 | Risk of Fetal Distress | Not analyzed | Irrelevant outcome |
| 10 | Risk of Preterm Delivery | Favors metformin | inconsistent |

**Supplementary Table 4.** Our NMA results in comparison to Yu 2021 (Yu et al., 2021)

| **No.** | **Outcome** | **Treatment favor** | **Consistency** |
| --- | --- | --- | --- |
| 1 | FBG | No difference | Consistent |
| 2 | 2h PG | No difference | Consistent |
| 3 | HbA1c | Not analyzed | Irrelevant outcome |
| 4 | Anomaly | Not analyzed | Irrelevant outcome |
| 5 | Hyperbiliru | Not analyzed | Irrelevant outcome |
| 6 | LGA | Favors glyburide | Inconsistent |
| 7 | Macrosomia | Not analyzed | Irrelevant outcome |
| 8 | NICU | Favors insulin | Inconsistent |
| 9 | Neonatal hypoglycemia | Favors glyburide | Consistent |
| 10 | Obstetric trauma | Not analyzed | Irrelevant outcome |
| 11 | Perinatal death | No difference | Consistent |
| 12 | Preterm birth | No difference | Consistent |
| 13 | RDS | Not analyzed | Irrelevant outcome |
| 14 | SGA | Not analyzed | Irrelevant outcome |
| 15 | Birth weight | Favors insulin and glyburide | Consistent |
| 16 | Preeclampsia | No difference | Consistent |

**Supplementary Table 5.** Search strategies

| Database |  |  |
| --- | --- | --- |
| Web of science |  |  |
| 1 | (((((((((ALL=(Diabetes, Gestational))) OR ALL=(Diabetes, Pregnancy-Induced)) OR ALL=( Diabetes, Pregnancy Induced)) OR ALL=(Pregnancy-Induced Diabetes)) OR ALL=(Gestational Diabetes))) OR ALL=(Diabetes Mellitus, Gestational)) OR ALL=(Gestational Diabetes Mellitus)) AND ((((ALL=(Randomized Controlled Trials)) OR ALL=(Clinical Trials, Randomized)) OR ALL=(Trials, Randomized Clinical)) OR ALL=(Controlled Clinical Trials, Randomized)) OR ALL=(RCT) AND (((((((((((ALL=(Insulin)) OR ALL=(Insulin, Regular)) OR ALL=(Regular Insulin)) OR ALL=(Soluble Insulin)) OR ALL=(Insulin, Soluble)) OR ALL=(Insulin A Chain)) OR ALL=(Sodium Insulin)) OR ALL=(Insulin, Sodium)) OR ALL=(Novolin)) OR ALL=(Iletin)) OR ALL=(Insulin B Chain)) AND ALL=(Chain, Insulin B) | 2 |
| 2 | (((((((((ALL=(Diabetes, Gestational))) OR ALL=(Diabetes, Pregnancy-Induced)) OR ALL=( Diabetes, Pregnancy Induced)) OR ALL=(Pregnancy-Induced Diabetes)) OR ALL=(Gestational Diabetes))) OR ALL=(Diabetes Mellitus, Gestational)) OR ALL=(Gestational Diabetes Mellitus)) AND ((((ALL=(Randomized Controlled Trials)) OR ALL=(Clinical Trials, Randomized)) OR ALL=(Trials, Randomized Clinical)) OR ALL=(Controlled Clinical Trials, Randomized)) OR ALL=(RCT) AND (((((((((((((((((((((((((((((ALL=(Hypoglycemic Agents)) OR ALL=(Agents, Hypoglycemic)) OR ALL=(Hypoglycemic Agent)) OR ALL=(Agent, Hypoglycemic)) OR ALL=(Antihyperglycemic Agent)) OR ALL=(Agent, Antihyperglycemic)) OR ALL=(Antihyperglycemics)) OR ALL=(Hypoglycemic)) OR ALL=(Hypoglycemic Drugs)) OR ALL=(Drugs, Hypoglycemic)) OR ALL=(Hypoglycemics)) OR ALL=(Antihyperglycemic Agents)) OR ALL=(Agents, Antihyperglycemic)) OR ALL=(Antihyperglycemic)) OR ALL=(Hypoglycemic Drug)) OR ALL=(Drug, Hypoglycemic)) OR ALL=(Antidiabetics)) OR ALL=(Antidiabetic Drug)) OR ALL=(Drug, Antidiabetic)) OR ALL=(Antidiabetic Drugs)) OR ALL=(Drugs, Antidiabetic)) OR ALL=(Antidiabetic Agents)) OR ALL=(Agents, Antidiabetic)) OR ALL=(Antidiabetic Agent)) OR ALL=(Agent, Antidiabetic)) OR ALL=(Antidiabetic)) OR ALL=(Hypoglycemic Effect)) OR ALL=(Effect, Hypoglycemic)) OR ALL=(Hypoglycemic Effects)) OR ALL=(Effects, Hypoglycemic) | 81 |
| 3 | (((((((((ALL=(Diabetes, Gestational))) OR ALL=(Diabetes, Pregnancy-Induced)) OR ALL=( Diabetes, Pregnancy Induced)) OR ALL=(Pregnancy-Induced Diabetes)) OR ALL=(Gestational Diabetes))) OR ALL=(Diabetes Mellitus, Gestational)) OR ALL=(Gestational Diabetes Mellitus)) AND ((((ALL=(Randomized Controlled Trials)) OR ALL=(Clinical Trials, Randomized)) OR ALL=(Trials, Randomized Clinical)) OR ALL=(Controlled Clinical Trials, Randomized)) OR ALL=(RCT) AND (((((((((((ALL=(Insulin, Isophane)) OR ALL=(Isophane Insulin)) OR ALL=(Neutral Protamine Hagedorn Insulin)) OR ALL=(Protamine Hagedorn Insulin)) OR ALL=(Hagedorn Insulin, Protamine)) OR ALL=(Isophane Insulin, Regular)) OR ALL=(Regular Isophane Insulin)) OR ALL=(Insulin, NPH)) OR ALL=(NPH Insulin)) OR ALL=(Insulin, Protamine Zinc)) OR ALL=(Protamine Zinc Insulin)) OR ALL=(Zinc Insulin, Protamine) | 27 |
| 4 | (((((((((ALL=(Diabetes, Gestational))) OR ALL=(Diabetes, Pregnancy-Induced)) OR ALL=( Diabetes, Pregnancy Induced)) OR ALL=(Pregnancy-Induced Diabetes)) OR ALL=(Gestational Diabetes))) OR ALL=(Diabetes Mellitus, Gestational)) OR ALL=(Gestational Diabetes Mellitus)) AND ((((ALL=(Randomized Controlled Trials)) OR ALL=(Clinical Trials, Randomized)) OR ALL=(Trials, Randomized Clinical)) OR ALL=(Controlled Clinical Trials, Randomized)) OR ALL=(RCT) AND (((((((((ALL=(Insulin Aspart)) OR ALL=(Aspart, Insulin)) OR ALL=(Insulin, Aspartic Acid(B28)-)) OR ALL=(Insulin B28asp)) OR ALL=(B28asp, Insulin)) OR ALL=(Insulin-Aspart)) OR ALL=(B28-Asp-Insulin)) OR ALL=(B28 Asp Insulin)) OR ALL=(NovoLog)) OR ALL=(NovoRapid) | 27 |
| 5 | (((((((((ALL=(Diabetes, Gestational))) OR ALL=(Diabetes, Pregnancy-Induced)) OR ALL=( Diabetes, Pregnancy Induced)) OR ALL=(Pregnancy-Induced Diabetes)) OR ALL=(Gestational Diabetes))) OR ALL=(Diabetes Mellitus, Gestational)) OR ALL=(Gestational Diabetes Mellitus)) AND ((((ALL=(Randomized Controlled Trials)) OR ALL=(Clinical Trials, Randomized)) OR ALL=(Trials, Randomized Clinical)) OR ALL=(Controlled Clinical Trials, Randomized)) OR ALL=(RCT) AND ((((((((((ALL=(Insulin Lispro)) OR ALL=(Lispro, Insulin)) OR ALL=(28(B)-Lys-29(B)-Pro-Insulin)) OR ALL=(28(B)-Lysine-29(B)-Prolineinsulin)) OR ALL=(Insulin, Lysyl(28B)-Prolyl(28B)-)) OR ALL=(Lispro)) OR ALL=(LYSPRO)) OR ALL=(Insulin, Lys(28B)-Pro(29B)-)) OR ALL=(Humalog Kwikpen)) OR ALL=(Kwikpen, Humalog)) OR ALL=(Humalog) | 21 |
| 6 | (((((((((ALL=(Diabetes, Gestational))) OR ALL=(Diabetes, Pregnancy-Induced)) OR ALL=( Diabetes, Pregnancy Induced)) OR ALL=(Pregnancy-Induced Diabetes)) OR ALL=(Gestational Diabetes))) OR ALL=(Diabetes Mellitus, Gestational)) OR ALL=(Gestational Diabetes Mellitus)) AND ((((ALL=(Randomized Controlled Trials)) OR ALL=(Clinical Trials, Randomized)) OR ALL=(Trials, Randomized Clinical)) OR ALL=(Controlled Clinical Trials, Randomized)) OR ALL=(RCT) AND ((((((((((((((ALL=(Insulin Detemir)) OR ALL=(Detemir, Insulin)) OR ALL=(Basal Insulin Detemir)) OR ALL=(Detemir, Basal Insulin)) OR ALL=(Insulin Detemir, Basal)) OR ALL=(NN304)) OR ALL=(NN-304)) OR ALL=(NN 304)) OR ALL=(B29-tetradecanoyl-Lys-B30-des-Ala-insulin)) OR ALL=(B29 tetradecanoyl Lys B30 des Ala insulin)) OR ALL=(Insulin, Tetradecanoyl-Lys(B29)-des-Ala(B30))) OR ALL=(12C-Lys(B29)-DB30I)) OR ALL=(Des-(B30)-insulin, Lys(B29)-tetradecanoyl)) OR ALL=(Insulin, Tetradecanoyllysyl(B29)-desalanyl(B30))) OR ALL=(Levemir) | 24 |
| 7 | (((((((((ALL=(Diabetes, Gestational))) OR ALL=(Diabetes, Pregnancy-Induced)) OR ALL=( Diabetes, Pregnancy Induced)) OR ALL=(Pregnancy-Induced Diabetes)) OR ALL=(Gestational Diabetes))) OR ALL=(Diabetes Mellitus, Gestational)) OR ALL=(Gestational Diabetes Mellitus)) AND ((((ALL=(Randomized Controlled Trials)) OR ALL=(Clinical Trials, Randomized)) OR ALL=(Trials, Randomized Clinical)) OR ALL=(Controlled Clinical Trials, Randomized)) OR ALL=(RCT) AND ((((((((((((((ALL=(Insulin Glargine)) OR ALL=(Glargine, Insulin)) OR ALL=(A21-Gly-B31-Arg-B32-Arg-insulin)) OR ALL=(A21 Gly B31 Arg B32 Arg insulin)) OR ALL=(Insulin, Glycyl(A21)-Arginyl(B31,B32))) OR ALL=(Glargine)) OR ALL=(Insulin, Gly(A21)-Arg(B31,B32))) OR ALL=(Lantus)) OR ALL=(Lantus Solostar)) OR ALL=(Solostar, Lantus)) OR ALL=(Basaglar)) OR ALL=(HOE 901)) OR ALL=(901, HOE)) OR ALL=(HOE-901)) OR ALL=(HOE901) | 20 |
| Remove duplicate |  | 76 |
| NCBI |  |  |
| 1 | (((((((((Diabetes, Gestational[MeSH Terms]) OR (Diabetes, Pregnancy-Induced[MeSH Terms])) OR (Diabetes, Pregnancy Induced[MeSH Terms])) OR (Pregnancy-Induced Diabetes[MeSH Terms])) OR (Gestational Diabetes[MeSH Terms])) OR (Diabetes Mellitus, Gestational[MeSH Terms])) OR (Gestational Diabetes Mellitus[MeSH Terms])) AND (((((((Diabetes, Gestational[MeSH Terms]) OR (Diabetes, Pregnancy-Induced[MeSH Terms])) OR (Diabetes, Pregnancy Induced[MeSH Terms])) OR (Pregnancy-Induced Diabetes[MeSH Terms])) OR (Gestational Diabetes[MeSH Terms])) OR (Diabetes Mellitus, Gestational[MeSH Terms])) OR (Gestational Diabetes Mellitus[MeSH Terms])))) AND ((((Randomized Controlled Trials) OR (Clinical Trials, Randomized)) OR (Trials, Randomized Clinical)) OR (Controlled Clinical Trials, Randomized)) OR (RCT) AND (((((((((((((((((((((((((((((Hypoglycemic Agents) OR (Agents, Hypoglycemic)) OR (Hypoglycemic Agent)) OR (Agent, Hypoglycemic)) OR (Antihyperglycemic Agent)) OR (Agent, Antihyperglycemic)) OR (Antihyperglycemics)) OR (Hypoglycemic)) OR (Hypoglycemic Drugs)) OR (Drugs, Hypoglycemic)) OR (Hypoglycemics)) OR (Antihyperglycemic Agents)) OR (Agents, Antihyperglycemic)) OR (Antihyperglycemic)) OR (Hypoglycemic Drug)) OR (Drug, Hypoglycemic)) OR (Antidiabetics)) OR (Antidiabetic Drug)) OR (Drug, Antidiabetic)) OR (Antidiabetic Drugs)) OR (Drugs, Antidiabetic)) OR (Antidiabetic Agents)) OR (Agents, Antidiabetic)) OR (Antidiabetic Agent)) OR (Agent, Antidiabetic)) OR (Antidiabetic)) OR (Hypoglycemic Effect)) OR (Effect, Hypoglycemic)) OR (Hypoglycemic Effects)) OR (Effects, Hypoglycemic) | 278 |
| 2 | (((((((((Diabetes, Gestational[MeSH Terms]) OR (Diabetes, Pregnancy-Induced[MeSH Terms])) OR (Diabetes, Pregnancy Induced[MeSH Terms])) OR (Pregnancy-Induced Diabetes[MeSH Terms])) OR (Gestational Diabetes[MeSH Terms])) OR (Diabetes Mellitus, Gestational[MeSH Terms])) OR (Gestational Diabetes Mellitus[MeSH Terms])) AND (((((((Diabetes, Gestational[MeSH Terms]) OR (Diabetes, Pregnancy-Induced[MeSH Terms])) OR (Diabetes, Pregnancy Induced[MeSH Terms])) OR (Pregnancy-Induced Diabetes[MeSH Terms])) OR (Gestational Diabetes[MeSH Terms])) OR (Diabetes Mellitus, Gestational[MeSH Terms])) OR (Gestational Diabetes Mellitus[MeSH Terms])))) AND ((((Randomized Controlled Trials) OR (Clinical Trials, Randomized)) OR (Trials, Randomized Clinical)) OR (Controlled Clinical Trials, Randomized)) OR (RCT) AND (((((((((((Insulin, Isophane) OR (Isophane Insulin)) OR (Neutral Protamine Hagedorn Insulin)) OR (Protamine Hagedorn Insulin)) OR (Hagedorn Insulin, Protamine)) OR (Isophane Insulin, Regular)) OR (Regular Isophane Insulin)) OR (Insulin, NPH)) OR (NPH Insulin)) OR (Insulin, Protamine Zinc)) OR (Protamine Zinc Insulin)) OR (Zinc Insulin, Protamine) | 13 |
| 3 | (((((((((Diabetes, Gestational[MeSH Terms]) OR (Diabetes, Pregnancy-Induced[MeSH Terms])) OR (Diabetes, Pregnancy Induced[MeSH Terms])) OR (Pregnancy-Induced Diabetes[MeSH Terms])) OR (Gestational Diabetes[MeSH Terms])) OR (Diabetes Mellitus, Gestational[MeSH Terms])) OR (Gestational Diabetes Mellitus[MeSH Terms])) AND (((((((Diabetes, Gestational[MeSH Terms]) OR (Diabetes, Pregnancy-Induced[MeSH Terms])) OR (Diabetes, Pregnancy Induced[MeSH Terms])) OR (Pregnancy-Induced Diabetes[MeSH Terms])) OR (Gestational Diabetes[MeSH Terms])) OR (Diabetes Mellitus, Gestational[MeSH Terms])) OR (Gestational Diabetes Mellitus[MeSH Terms])))) AND ((((Randomized Controlled Trials) OR (Clinical Trials, Randomized)) OR (Trials, Randomized Clinical)) OR (Controlled Clinical Trials, Randomized)) OR (RCT) AND (((((((((Insulin Aspart) OR (Aspart, Insulin)) OR (Insulin, Aspartic Acid(B28)-)) OR (Insulin B28asp)) OR (B28asp, Insulin)) OR (Insulin-Aspart)) OR (B28-Asp-Insulin)) OR (B28 Asp Insulin)) OR (NovoLog)) OR (NovoRapid) | 12 |
| 4 | (((((((((Diabetes, Gestational[MeSH Terms]) OR (Diabetes, Pregnancy-Induced[MeSH Terms])) OR (Diabetes, Pregnancy Induced[MeSH Terms])) OR (Pregnancy-Induced Diabetes[MeSH Terms])) OR (Gestational Diabetes[MeSH Terms])) OR (Diabetes Mellitus, Gestational[MeSH Terms])) OR (Gestational Diabetes Mellitus[MeSH Terms])) AND (((((((Diabetes, Gestational[MeSH Terms]) OR (Diabetes, Pregnancy-Induced[MeSH Terms])) OR (Diabetes, Pregnancy Induced[MeSH Terms])) OR (Pregnancy-Induced Diabetes[MeSH Terms])) OR (Gestational Diabetes[MeSH Terms])) OR (Diabetes Mellitus, Gestational[MeSH Terms])) OR (Gestational Diabetes Mellitus[MeSH Terms])))) AND ((((Randomized Controlled Trials) OR (Clinical Trials, Randomized)) OR (Trials, Randomized Clinical)) OR (Controlled Clinical Trials, Randomized)) OR (RCT) AND ((((((((((Insulin Lispro) OR (Lispro, Insulin)) OR (28(B)-Lys-29(B)-Pro-Insulin)) OR (28(B)-Lysine-29(B)-Prolineinsulin)) OR (Insulin, Lysyl(28B)-Prolyl(28B)-)) OR (Lispro)) OR (LYSPRO)) OR (Insulin, Lys(28B)-Pro(29B)-)) OR (Humalog Kwikpen)) OR (Kwikpen, Humalog)) OR (Humalog) | 8 |
| 5 | (((((((((Diabetes, Gestational[MeSH Terms]) OR (Diabetes, Pregnancy-Induced[MeSH Terms])) OR (Diabetes, Pregnancy Induced[MeSH Terms])) OR (Pregnancy-Induced Diabetes[MeSH Terms])) OR (Gestational Diabetes[MeSH Terms])) OR (Diabetes Mellitus, Gestational[MeSH Terms])) OR (Gestational Diabetes Mellitus[MeSH Terms])) AND (((((((Diabetes, Gestational[MeSH Terms]) OR (Diabetes, Pregnancy-Induced[MeSH Terms])) OR (Diabetes, Pregnancy Induced[MeSH Terms])) OR (Pregnancy-Induced Diabetes[MeSH Terms])) OR (Gestational Diabetes[MeSH Terms])) OR (Diabetes Mellitus, Gestational[MeSH Terms])) OR (Gestational Diabetes Mellitus[MeSH Terms])))) AND ((((Randomized Controlled Trials) OR (Clinical Trials, Randomized)) OR (Trials, Randomized Clinical)) OR (Controlled Clinical Trials, Randomized)) OR (RCT) AND ((((((((((((((Insulin Detemir) OR (Detemir, Insulin)) OR (Basal Insulin Detemir)) OR (Detemir, Basal Insulin)) OR (Insulin Detemir, Basal)) OR (NN304)) OR (NN-304)) OR (NN 304)) OR (B29-tetradecanoyl-Lys-B30-des-Ala-insulin)) OR (B29 tetradecanoyl Lys B30 des Ala insulin)) OR (Insulin, Tetradecanoyl-Lys(B29)-des-Ala(B30))) OR (12C-Lys(B29)-DB30I)) OR (Des-(B30)-insulin, Lys(B29)-tetradecanoyl)) OR (Insulin, Tetradecanoyllysyl(B29)-desalanyl(B30))) OR (Levemir) | 6 |
| 6 | (((((((((Diabetes, Gestational[MeSH Terms]) OR (Diabetes, Pregnancy-Induced[MeSH Terms])) OR (Diabetes, Pregnancy Induced[MeSH Terms])) OR (Pregnancy-Induced Diabetes[MeSH Terms])) OR (Gestational Diabetes[MeSH Terms])) OR (Diabetes Mellitus, Gestational[MeSH Terms])) OR (Gestational Diabetes Mellitus[MeSH Terms])) AND (((((((Diabetes, Gestational[MeSH Terms]) OR (Diabetes, Pregnancy-Induced[MeSH Terms])) OR (Diabetes, Pregnancy Induced[MeSH Terms])) OR (Pregnancy-Induced Diabetes[MeSH Terms])) OR (Gestational Diabetes[MeSH Terms])) OR (Diabetes Mellitus, Gestational[MeSH Terms])) OR (Gestational Diabetes Mellitus[MeSH Terms])))) AND ((((Randomized Controlled Trials) OR (Clinical Trials, Randomized)) OR (Trials, Randomized Clinical)) OR (Controlled Clinical Trials, Randomized)) OR (RCT) AND ((((((((((((((Insulin Glargine) OR (Glargine, Insulin)) OR (A21-Gly-B31-Arg-B32-Arg-insulin)) OR (A21 Gly B31 Arg B32 Arg insulin)) OR (Insulin, Glycyl(A21)-Arginyl(B31,B32))) OR (Glargine)) OR (Insulin, Gly(A21)-Arg(B31,B32))) OR (Lantus)) OR (Lantus Solostar)) OR (Solostar, Lantus)) OR (Basaglar)) OR (HOE 901)) OR (901, HOE)) OR (HOE-901)) OR (HOE901) | 1 |
| Remove duplicate |  | 91 |
| Embase |  |  |
| 1 | ('pregnancy diabetes mellitus'/exp OR 'diabetes, gestational':ti,ab,kw OR 'diabetes, pregnancy-induced':ti,ab,kw OR 'diabetes, pregnancy induced':ti,ab,kw OR 'pregnancy-induced diabetes':ti,ab,kw OR 'gestational diabetes':ti,ab,kw OR 'diabetes mellitus, gestational':ti,ab,kw OR 'gestational diabetes mellitus':ti,ab,kw) AND ('randomized controlled trial (topic)'/exp OR 'randomized controlled trials':ti,ab,kw OR 'clinical trials, randomized':ti,ab,kw OR 'trials, randomized clinical':ti,ab,kw OR 'controlled clinical trials, randomized':ti,ab,kw OR rct:ti,ab,kw) AND ('human insulin'/exp OR insulin:ti,ab,kw OR 'insulin, regular':ti,ab,kw OR 'regular insulin':ti,ab,kw OR 'soluble insulin':ti,ab,kw OR 'insulin, soluble':ti,ab,kw OR 'insulin a chain':ti,ab,kw OR 'sodium insulin':ti,ab,kw OR 'insulin, sodium' OR (('insulin,'/exp OR insulin,) AND ('sodium'/exp OR sodium)) OR 'novolin'/exp OR novolin OR 'iletin'/exp OR iletin OR 'insulin b chain'/exp OR 'insulin b chain' OR (('insulin'/exp OR insulin) AND b AND chain) OR 'chain, insulin b' OR (chain, AND ('insulin'/exp OR insulin) AND b) | 470 |
| 2 | ('pregnancy diabetes mellitus'/exp OR 'diabetes, gestational':ti,ab,kw OR 'diabetes, pregnancy-induced':ti,ab,kw OR 'diabetes, pregnancy induced':ti,ab,kw OR 'pregnancy-induced diabetes':ti,ab,kw OR 'gestational diabetes':ti,ab,kw OR 'diabetes mellitus, gestational':ti,ab,kw OR 'gestational diabetes mellitus':ti,ab,kw) AND ('randomized controlled trial (topic)'/exp OR 'randomized controlled trials':ti,ab,kw OR 'clinical trials, randomized':ti,ab,kw OR 'trials, randomized clinical':ti,ab,kw OR 'controlled clinical trials, randomized':ti,ab,kw OR rct:ti,ab,kw) AND ('isophane insulin'/exp OR 'insulin, isophane':ti,ab,kw OR 'isophane insulin':ti,ab,kw OR 'neutral protamine hagedorn insulin':ti,ab,kw OR 'protamine hagedorn insulin':ti,ab,kw OR 'hagedorn insulin, protamine':ti,ab,kw OR 'isophane insulin, regular':ti,ab,kw OR 'regular isophane insulin':ti,ab,kw OR 'insulin, nph'/exp OR 'insulin, nph' OR (('insulin,'/exp OR insulin,) AND ('nph'/exp OR nph)) OR 'nph insulin'/exp OR 'nph insulin' OR (('nph'/exp OR nph) AND ('insulin'/exp OR insulin)) OR 'insulin, protamine zinc'/exp OR 'insulin, protamine zinc' OR (('insulin,'/exp OR insulin,) AND ('protamine'/exp OR protamine) AND ('zinc'/exp OR zinc)) OR 'protamine zinc insulin'/exp OR 'protamine zinc insulin' OR (('protamine'/exp OR protamine) AND ('zinc'/exp OR zinc) AND ('insulin'/exp OR insulin)) OR 'zinc insulin, protamine' OR (('zinc'/exp OR zinc) AND ('insulin,'/exp OR insulin,) AND ('protamine'/exp OR protamine)) | 38 |
| 3 | ('pregnancy diabetes mellitus'/exp OR 'diabetes, gestational':ti,ab,kw OR 'diabetes, pregnancy-induced':ti,ab,kw OR 'diabetes, pregnancy induced':ti,ab,kw OR 'pregnancy-induced diabetes':ti,ab,kw OR 'gestational diabetes':ti,ab,kw OR 'diabetes mellitus, gestational':ti,ab,kw OR 'gestational diabetes mellitus':ti,ab,kw) AND ('randomized controlled trial (topic)'/exp OR 'randomized controlled trials':ti,ab,kw OR 'clinical trials, randomized':ti,ab,kw OR 'trials, randomized clinical':ti,ab,kw OR 'controlled clinical trials, randomized':ti,ab,kw OR rct:ti,ab,kw) AND ('insulin lispro'/exp OR 'insulin lispro':ti,ab,kw OR 'lispro, insulin':ti,ab,kw OR (28:ti,ab,kw AND 'lys 29':ti,ab,kw AND b:ti,ab,kw AND 'pro insulin':ti,ab,kw) OR (28:ti,ab,kw AND 'lysine 29':ti,ab,kw AND b:ti,ab,kw AND -prolineinsulin:ti,ab,kw) OR ('insulin, lysyl':ti,ab,kw AND -prolyl:ti,ab,kw AND 28b:ti,ab,kw AND -:ti,ab,kw) OR lispro:ti,ab,kw OR lyspro:ti,ab,kw OR ('insulin, lys':ti,ab,kw AND 28b:ti,ab,kw AND -pro:ti,ab,kw AND 29b:ti,ab,kw AND -:ti,ab,kw) OR 'humalog kwikpen':ti,ab,kw OR 'kwikpen, humalog':ti,ab,kw OR humalog:ti,ab,kw) | 36 |
| 4 | ('pregnancy diabetes mellitus'/exp OR 'diabetes, gestational':ti,ab,kw OR 'diabetes, pregnancy-induced':ti,ab,kw OR 'diabetes, pregnancy induced':ti,ab,kw OR 'pregnancy-induced diabetes':ti,ab,kw OR 'gestational diabetes':ti,ab,kw OR 'diabetes mellitus, gestational':ti,ab,kw OR 'gestational diabetes mellitus':ti,ab,kw) AND ('randomized controlled trial (topic)'/exp OR 'randomized controlled trials':ti,ab,kw OR 'clinical trials, randomized':ti,ab,kw OR 'trials, randomized clinical':ti,ab,kw OR 'controlled clinical trials, randomized':ti,ab,kw OR rct:ti,ab,kw) AND ('insulin detemir'/exp OR 'insulin detemir':ti,ab,kw OR 'detemir, insulin':ti,ab,kw OR 'basal insulin detemir':ti,ab,kw OR 'detemir, basal insulin':ti,ab,kw OR 'insulin detemir, basal':ti,ab,kw OR nn304:ti,ab,kw OR 'nn 304':ti,ab,kw OR 'b29 tetradecanoyl lys b30 des ala insulin':ti,ab,kw OR ('insulin, tetradecanoyl-lys':ti,ab,kw AND b29:ti,ab,kw AND 'des ala':ti,ab,kw AND b30:ti,ab,kw) OR ('12c lys':ti,ab,kw AND b29:ti,ab,kw AND -db30i:ti,ab,kw) OR (des-:ti,ab,kw AND b30:ti,ab,kw AND '-insulin, lys':ti,ab,kw AND b29:ti,ab,kw AND -tetradecanoyl:ti,ab,kw) OR ('insulin, tetradecanoyllysyl':ti,ab,kw AND b29:ti,ab,kw AND -desalanyl:ti,ab,kw AND b30:ti,ab,kw) OR levemir:ti,ab,kw) | 40 |
| 5 | ('pregnancy diabetes mellitus'/exp OR 'diabetes, gestational':ti,ab,kw OR 'diabetes, pregnancy-induced':ti,ab,kw OR 'diabetes, pregnancy induced':ti,ab,kw OR 'pregnancy-induced diabetes':ti,ab,kw OR 'gestational diabetes':ti,ab,kw OR 'diabetes mellitus, gestational':ti,ab,kw OR 'gestational diabetes mellitus':ti,ab,kw) AND ('randomized controlled trial (topic)'/exp OR 'randomized controlled trials':ti,ab,kw OR 'clinical trials, randomized':ti,ab,kw OR 'trials, randomized clinical':ti,ab,kw OR 'controlled clinical trials, randomized':ti,ab,kw OR rct:ti,ab,kw) AND ('insulin glargine'/exp OR 'insulin glargine':ti,ab,kw OR 'glargine, insulin':ti,ab,kw OR 'a21 gly b31 arg b32 arg insulin':ti,ab,kw OR ('insulin, glycyl':ti,ab,kw AND a21:ti,ab,kw AND -arginyl:ti,ab,kw AND b31,b32:ti,ab,kw) OR glargine:ti,ab,kw OR ('insulin, gly':ti,ab,kw AND a21:ti,ab,kw AND -arg:ti,ab,kw AND b31,b32:ti,ab,kw) OR lantus:ti,ab,kw OR 'lantus solostar':ti,ab,kw OR 'solostar, lantus':ti,ab,kw OR basaglar:ti,ab,kw OR '901, hoe':ti,ab,kw OR 'hoe 901':ti,ab,kw OR hoe901:ti,ab,kw) | 28 |
| 6 | ('pregnancy diabetes mellitus'/exp OR 'diabetes, gestational':ti,ab,kw OR 'diabetes, pregnancy-induced':ti,ab,kw OR 'diabetes, pregnancy induced':ti,ab,kw OR 'pregnancy-induced diabetes':ti,ab,kw OR 'gestational diabetes':ti,ab,kw OR 'diabetes mellitus, gestational':ti,ab,kw OR 'gestational diabetes mellitus':ti,ab,kw) AND ('randomized controlled trial (topic)'/exp OR 'randomized controlled trials':ti,ab,kw OR 'clinical trials, randomized':ti,ab,kw OR 'trials, randomized clinical':ti,ab,kw OR 'controlled clinical trials, randomized':ti,ab,kw OR rct:ti,ab,kw) AND ('antidiabetic agent'/exp OR 'hypoglycemic agents':ti,ab,kw OR 'agents, hypoglycemic':ti,ab,kw OR 'hypoglycemic agent':ti,ab,kw OR 'agent, hypoglycemic':ti,ab,kw OR 'antihyperglycemic agent':ti,ab,kw OR 'agent, antihyperglycemic':ti,ab,kw OR antihyperglycemics:ti,ab,kw OR hypoglycemic:ti,ab,kw OR 'hypoglycemic drugs':ti,ab,kw OR 'drugs, hypoglycemic':ti,ab,kw OR hypoglycemics:ti,ab,kw OR 'antihyperglycemic agents':ti,ab,kw OR 'agents, antihyperglycemic':ti,ab,kw OR antihyperglycemic:ti,ab,kw OR 'hypoglycemic drug':ti,ab,kw OR 'drug, hypoglycemic':ti,ab,kw OR antidiabetics:ti,ab,kw OR 'antidiabetic drug':ti,ab,kw OR 'drug, antidiabetic':ti,ab,kw OR 'antidiabetic drugs':ti,ab,kw OR 'drugs, antidiabetic':ti,ab,kw OR 'antidiabetic agents':ti,ab,kw OR 'agents, antidiabetic':ti,ab,kw OR 'antidiabetic agent':ti,ab,kw OR 'agent, antidiabetic':ti,ab,kw OR antidiabetic:ti,ab,kw OR 'hypoglycemic effect':ti,ab,kw OR 'effect, hypoglycemic':ti,ab,kw OR 'hypoglycemic effects':ti,ab,kw OR 'effects, hypoglycemic':ti,ab,kw) | 470 |
| Remove duplicate |  | 538 |
| Total |  | 705 |

**Supplementary table 6.** The baseline data of the included RCTs

| **Author year** | **Country** | **Setting, period, criteria** | **Study design/participants** | **Groups** | **Diagnose（OGTT）** | **Pre-intervention** | **Target blood glucose** |
| --- | --- | --- | --- | --- | --- | --- | --- |
| Ainuddin 2015(Ainuddin et al., 2015) | Pakistan | **Settin**g: Prenatal clinics at Lyari General Hospital, Karachi and Mamji Hospital, Karachi.  **Period:** December 2008 to December 2010  **Criteria:** Women included in the study were between 20 to 46 years of age and had GDM diagnosed with a singleton pregnancy between 20 and 36 weeks of gestation. | Open label parallel RCT | Metformin (43), insulin (75) | ADA2003  Fasting ≥5.3 mmol/l, 1h ≥10 mmol/l ， 2h≥8.6 mmol/l | Dietary and life style modifications. | Fasting ＜100 mg/dl (5.5 mmol/l) and RBS (post parandial) ＜126 mg/dl (7 mmol/l) |
| Tertti 2013(Tertti et al., 2013) | Finland | **Settin**g: Turku university central hospital  **Period:** June 2006 and December 2010  **Criteria:** Women with singleton pregnancy. | Open label parallel RCT | Metformin (110), insulin (107) | Finnish national criteria  Before update: fasting ≥4.8 mmol/l, 1 h ≥ 10.0 mmol/l and 2 h ≥ 8.7 mmol/l  After update fasting ≥5.3, 1h ≥10.0 and 2h ≥8.6 mmol/l, respectively | Not given | Target values for fasting <5.5 mmol/l after overnight fast and <7.8 mmol/l 60 min after meals. |
| Spaulonci 2013(Spaulonci et al., 2013) | Brazil | **Setting**: Hospital das clinics, Sao Paulo University School of Medicine  **Period:** Nov. 1, 2007, and Jan 31, 2010  **Criteria:** singleton pregnancy, use of diet and exercise for a minimum period of 1 week without satisfactory glycemic control, absence of risk factors for lactic acidosis | Prospective comparative study | Metformin (47), insulin (47) | ADA criteria 2011  Fasting ≥5.2 mmol/l, 1 h ≥ 10.0 mmol/l and 2 h ≥7.7 mmol/l. | Diet and exercise. | Fasting ≤95 mg/dL and 2 hours after a meal ≤120 mg/dL. |
| Anjalakshi 2007(Anjalakshi et al., 2007) | India | **Setting:** Hospital of Government Stanley Medical College, Dr. V. Seshiah Diabetes Care and Research Institute  **Period:** -  **Criteria:** Consecutive pregnant women attending the antenatal clinic diagnosed as GDM | RCT | Glyburide (10), insulin (13) | WHO criteria  75-g OGTT fasting plasma glucose ≥ 7.0 mmol/L; 2-hour post 75-g oral glucose load ≥ 7.8mmol/L | Medical Nutrition Therapy | 2 h PG＜ 10.0 mmol/l |
| Arshad 2017(Arshad et al., 2017) | [Pakistan](javascript:;) | **Setting:** Lyari General Hospital, Mamji Hospital and Civil Hospital  **Period:** 2010-2012  **Criteria:** Bad obstetric history | RCT | Metformin (25), insulin (25) | WHO criteria  Fasting plasma glucose ≥ 5.1-6.9mmol/L; 1-hour post 75-g oral glucose load ≥ 10mmol/L; 2-hour post 75-g oral glucose load ≥ 8.5-11.0mmol/L | Diet control and exercise | None |
| Ashoush 2016(Ashoush et al., 2016) | Egypt | **Setting:** antenatal care clinics of Ain Shams University Maternity Hospital  **Period:** January to November 2014  **Criteria:** singleton pregnancy, failure of satisfactory glycemic control despite adequate diet and exercise for at least 1 week after diagnosis absence of all of the following: fetal anomalies on ultrasonography, pregnancy complications apart from GDM, known intolerance to metformin or risk factors for lactic acidosis (renal or cardiac failure, severe hepatic or pulmonary disease) | Open-label, prospective RCT | Metformin (47), insulin (48) | ADA 2004  Fasting plasma glucose ≥ 5.3mmol/L; 1-hour post 75-g oral glucose load ≥ 10mmol/L; 2-hour post 75-g oral glucose load ≥ 8.6 mmol/L | Failing diet control | FBG＜100mg/dl， 2h after meals＜140mg/dl |
| Behrashi 2016(Behrashi et al., 2016) | Iran | **Setting**: Gynecology clinics of Shabihkhani and Shahid Beheshti Hospital of Kashan  **Period**: -  **Criteria**: 18–45 years, 11–33 weeks of gestation, absence of diabetes before pregnancy, singleton pregnancy, absence of known kidney, and hepatic, hematological, and/or cardiovascular disease | RCT | Glyburide（120），insulin (129) | Not IADPSG (Obstetrics. 24th ed. New York: McGraw Hill; 2014)  100g glucose OGTT FBS >95, 1 h >180, 2 h >155, and 3 h >140 mg/dl. | Exercise and diet | FBG＜90mg/dl， 2h after meals＜120 mg/dl |
| Bertini 2005(Bertini et al., 2005) | Brazil | **Setting**: Darcy Vargas Maternity  **Period:** October 1st 2003 to July 1st 2004  **Criteria:** Patients’ gestational age ranged at diagnosis from 11 to 33 weeks, with single gestations. | Open-label RCT | Insulin (27), glyburide (24) | WHO criteria 2002  75 g of glucose OGTT FBG≥110 mg/dL, 2h≥140 mg/dL | Diet and exercise | FBG＜90mg/dl， 2h after meals＜100 mg/dl |
| Feghali 2021(Feghali et al., 2021) | USA | **Setting:** Magee-Womens Hospital  **Period:** January 2009 to October 2012  **Criteria:** Women with singleton gestations and GDM. | RCT | Medical nutritional therapy (MNT) (293), glyburide (421), and insulin (102) | Not IADPSG (Carpenter-Coustan Criteria Am J Obstet Gynecol. 1982)  Pregnant women were screened using a 50 g, 1 h oral glucose tolerance test (OGTT) followed by a 100 g, 3 hour OGTT for screens greater than 135 mg/ dL. Two abnormal values (95, 180, 155, and 140 mg/dL), an elevated fasting value on 3 h OGTT, or 1 h OGTT greater than 200 mg/dL was diagnostic. | Nutritional counseling and education about recommended weight gain based on their pre-pregnancy BMI, clinical management of GDM, medical nutritional therapy counseling, carbohydrate-controlled diet | FBS＜95 mg/dL, 1h post-prandial＜140 mg/dL |
| George 2015(George et al., 2015) | Indian | **Setting:** Christian Medical College  **Period:** 2007 to 2010  **Criteria:** Pregnant women from 20–33 weeks gestation, fasting glucose ≥5.5 mmol/l and ≤7.2 mmol/l- and/or 2-h post prandial value ≥ 6.7 mmol/l and ≤13.9 mmol/l after MNT. | RCT | Glyburide (80), metformin (79) | National Diabetes Data group 1979.  FBG ≥5.3 mmol/l, 1 h ≥10 mmol/l, 2 h ≥8.6 mmol/ l, and 3 h≥7.8 mmol/l. National Diabetes Data group | Medical nutritional therapy (MNT) | FBS ≤5.3 mmol/l and 2-h postprandial level 6.7 mmol/l had to be achieved in 2–3 weeks |
| Ghomian 2018(Ghomian et al., 2019) | Iran | **Setting:** Three academic hospitals affiliated with Mashhad University of Medical Sciences.  **Period: -**  **Criteria:** Pregnant women aged between 18 and 40 with a gestational age over 24 weeks diagnosed with GDM, singleton pregnancy, failure to achieve glycemic control with exercise and diet during 1 week, absence of overt diabetes mellitus, absence of lactic acidosis risk factor, absence of fetal anomaly, absence of medical diseases in mothers such as kidney or liver diseases, and filling the informed consent for participation in the study. | RCT | metformin (143), insulin (143) | IADPSG  Fasting plasma glucose (FPG) levels exceed 105 mg/dl on SMBG or 1‐hr PG exceeds 155 mg/dl and 2‐hr PG exceeds 130 mg/dl. | Diet and Exercise regimens for 1 week | FPG and their 2‐hr FBG still remained above 95 and 120 mg/dl 2-h postprandial. |
| Hassan 2012(Hassan et al., 2012) | Pakistan | **Setting:** Dow University of Health Sciences and private maternity hospitals.  **Period:** December 12, 2008 to December 20, 2010  **Criteria:** The patients were selected from those attending the antenatal clinics and diagnosed with GDM after screening in antenatal clinic due to presence of high risk factors for diabetes mellitus. | RCT | Metformin (75), insulin (75) | WHO  At least two out of three abnormal high plasma glucose levels in a 75 gm OGTT FBS >95 mg/dl, 1 hour ≥ 180 mg/dl, 2 hour ≥ 155 mg/dl | Diet and exercise | FBS＜100mg/dl, post parandial of ＜126mg/dl |
| Ijas 2011(Ijäs et al., 2011) | Finland | **Setting:** Maternity outpatient clinics in a secondary and tertiary level hospital in Finland.  **Period:** 22 June 2005 and 30 June 2009  **Criteria:** Women with singleton pregnancies diagnosed with GDM between 12 and 34 weeks of gestation were asked to participate in the study. | Open-label prospective RCT | Metformin (47), insulin (50) | Not IADPSG  75-g oral glucose tolerance test (OGTT) after an overnight fast of 10 hours. The cut-off values for capillary plasma glucose concentrations were 5.3 (0 hour), 11.0 (1 hour) and 9.6 (2 hours) mmol/l. | Dietary and lifestyle counselling. Home monitoring of glucose concentrations twice a week by 4- to 6-point daily profiles | FBG＜5.3mmol/L, 2h after meals ＜6.7mmol/L |
| Lain 2009(Lain et al., 2009) | USA | **Setting:** Magee Women Hospital  **Period:** 2002-2005  **Criteria:** 24-34 weeks’ gestational age, a singleton pregnancy, no known fetal anomalies or intrauterine growth retardation, and no use of other medications with known glycemic effect. | RCT | Insulin (41) Glyburide (41) | Not IADPSG (Carpenter-Coustan Criteria Am J Obstet Gynecol. 1982)  Pregnant women were screened using a 50 g, 1 hour oral glucose tolerance test (OGTT) followed by a 100 g, 3 hour OGTT for screens greater than 135 mg/ dL. Two abnormal values (95, 180, 155, and 140 mg/dL), an elevated fasting value on 3 h OGTT, or 1 h OGTT greater than 200 mg/dL was diagnostic. | Education about diabetes dietary and blood sugar monitoring instructions | FBS ＜95mg/dl and ＜120 mg/dL (2 hour postprandial value) |
| Langer 2000(Langer et al., 2000) | USA | **Setting**: St. Luke’s–Roosevelt Hospital Center， University of Texas Health Science Center at San Antonio  **Period**: -  **Criteria**: singleton pregnancies and gestational diabetes | RCT | Insulin (203), Glyburide (201) | Not IADPGG (Carpenter-Coustan Criteria Am J Obstet Gynecol. 1982)  Pregnant women were screened using a 50 g, 1 hour oral glucose tolerance test (OGTT) followed by a 100 g, 3 hour OGTT for screens greater than 135 mg/ dL. Two abnormal values (95, 180, 155, and 140 mg/dL), an elevated fasting value on 3 h OGTT, or 1 h OGTT greater than 200 mg/dL was diagnostic. | Diet | A mean blood glucose concentration of 90 to 105 mg/dl, FBG 60 to 90 mg/dl, a preprandial blood glucose concentration of 80 to 95 mg/dl, and a postprandial blood glucose concentration of less than 120 mg/dl |
| Miriam 2015(Miriam George Fenn, 2015) | India | **Setting**: MOSC Medical College  **Period**:-  **Criteria**: GDM women | Prospective double blind RCT | Insulin (23), Glyburide (23) | Not IADPSG  Women who had plasma glucose concentrations above 130 mg per deciliter (7.3 mmol per liter) at one hour underwent a 100-g oral glucose-tolerance test. Women with two or more abnormal plasma glucose values were given a diagnosis of gestational diabetes. | Not given | Not given |
| Mirzamoradi 2015(Mirzamoradi et al., 2015) | Iran | **Setting**: Mahdieh Hospital  **Period**: March 2012-March 2013  **Criteria**: aged between 18-45 years with singleton pregnancies and in their 24-36 weeks of gestation | RCT | Insulin (59), Glyburide (37) | Not IADPSG  FBG >95 mg/dl, first-hour post-prandial blood glucose level was > 180 mg/dl or second hour post-prandial glucose level was >150 mg/dl. | Diet and nutrition clinic | FBG＜90mg/dl， 2h after meals＜120mg/dl |
| Mohammad 2019(Mohammad Galal, 2019) | Egypt | **Setting**: Al-Azhar University Hospital  **Period**: January, 2017 to October, 2018  **Criteria**: GDM women | Comparative prospective RCT | Metformin (50), insulin (50) | IADPSG  FBG≥92 mg/dl, 1 h≥180 mg/dl, 2 h ≥153 mg/dl. | Diet | FBG <95 mg/dL and 1 h post prandial <140 mg/dL, 2 h post prandial <120 mg/dL. |
| Moore 2010(Moore et al., 2010) | Mexico | **Setting**: The University of New Mexico in Albuquerque  **Period**: July 2003 to May 2008  **Criteria**: Women who did not maintain fasting blood glucose less than 105 mg/dL or 2-hour postprandial blood glucose less than 120 mg/dL with no other exclusion criteria were offered participation in the study. | RCT | Glyburide (74) Metformin (75) | Not IADPSG (Carpenter-Coustan Criteria Am J Obstet Gynecol. 1998)  Pregnant women receiving prenatal care were screened using a 50-g glucose load. Women with a 1-hour glucose level of 130 mg/dL or more were then given a 3-hour 100-g glucose tolerance test. | Counseling on diet and exercise and were initially treated with diet. | FBG＜105 mg/dL or 2-hour postprandial blood glucose＜120 mg/dL |
| Nachum 2017(Nachum et al., 2017) | Israel | **Setting**: Emek Medical center  **Period**: 5 January 2012 to 6 June 2014  **Criteria**: women between the ages of 18 and 45 years with GDM diagnosed between 13 and 33 weeks gestation and who required medical therapy because of poor glycemic control with diet alone. | Open-label parallel-group RCT | Glyburide (53) Metformin (51) | Not IADPSG (Carpenter-Coustan Criteria Am J Obstet Gynecol. 1979)  Pregnant women receiving prenatal care were screened using a 50-g glucose load. Women with a 1-hour glucose level of 130 mg/dL or more were then given a 3-hour 100-g glucose tolerance test. | Diet | Preprandial glucose values＜95 mg/dL, repeated postprandial values＜130 mg/dL |
| Niromanesh 2012(Niromanesh et al., 2012) | Iran | **Setting**: The Shariati Hospital and the Valiasr Hospital  **Period**: December 2010 and January 2012  **Criteria**: GDM women with singleton pregnancy and gestational age between 20 and 34 weeks who did not achieve glycemic control on diet | Single-blind RCT | Metformin (80) or insulin (80) | Not IADPSG (Carpenter-Coustan Criteria Am J Obstet Gynecol. 1979)  Pregnant women receiving prenatal care were screened using a 50-g glucose load. Women with a 1-hour glucose level of 130 mg/dL or more were then given a 3-hour 100-g glucose tolerance test. | Diet and regular physical exercise | FBG＜95 mg/dl or 2 h postprandial blood glucose＜120 mg/dl. |
| Picón-César 2021(Picón-César et al., 2021) | Spain | **Setting**: the hospitals Regional Universitario and Clínico Universitario Virgen de la Victoria.  **Period**: October 2016 and June 2019  **Criteria**: singleton pregnancy, age of 18-45 years, and gestational age (GA) of 14 to 35 weeks. | Prospective RCT | Metformin（100）, insulin (100) | Not IADPSG (ADA 1979)  A 50-gram oral glucose screening (O’Sullivan test) was followed by a 100-gram oral glucose tolerance test (OGTT) using the National Diabetes Data Group criteria. Isolated fasting glycemia at 100 mg/dL (5.6 mmol/L) was also considered as GDM. | Lifestyle changes and self-blood glucose monitoring (SBGM) 4 times a day | FBG 70 to 95 mg/dL and ≤140 mg/dl 1h after meals. |
| Rowan 2008(Rowan et al., 2008) | New Zealand and Australian | **Setting**: 10 New Zealand and Australian urban obstetrical hospitals.  **Period**: October 2002 to November 2006  **Criteria**: Between 18-45 years of age, had received a diagnosis of gestational diabetes mellitus according to the criteria of the Australasian Diabetes in Pregnancy Society (ADIPS),25 were pregnant with a single fetus between 20 and 33 weeks of gestation. | Open-label RCT | Metformin (363) Insulin (370) | NOT IADPSG(ADIPS)  FBG≥5.5.mmol/L, 2 h postprandial blood glucose≥8.0mmol/L. |  | FBG＜5.5mmol/L, 2-hour postprandial:＜7.0mmol/L |
| Ruholamin 2014(Ruholamin et al., 2014) | Iran | **Setting**: the Alzahra and Shahid beheshti Hospitals  **Period**: 2011  **Criteria**: 18-45 years aged; Pregnancy with a single fetus between 24 and 33 weeks of gestation; Current diagnosis of GDM according to the criteria of the Australasian Diabetes in Pregnancy Society; No response to lifestyle modification (diet and exercise) after 1 week; Written informed consent | RCT | Metformin (59) Insulin (60) | NOT IADPSG (Australasian Diabetes in Pregnancy Society 2000) | Diet | FBS＜95mg/dl, BS 2h＜120mg/dl |
| Saleh 2016(Saleh et al., 2016) | Egypt | **Setting**: Zagazig University Hospitals  **Period**: November 2012 to December 2014  **Criteria**: pregnancies had been complicated by GDM and did not respond to diet modifications or nutritional instructions alone in 3 weeks | Prospective RCT | Insulin (75), metformin (75) | NOT IADPSG (ADA criteria 2003)  ＞95 mg/dL (5.3 mmol/L), ＞180 mg/dL (10.0 mmol/L), and ＞155 mg/dL (8.6 mmol/L) | Diet | FBS＜5.5mmol/L, postprandial blood sugar (PPBS) levels at＜7mmol/L |
| Sénat 2018(Sénat et al., 2018) | France | **Setting**: (the Insulin Daonil trial [INDAO]) in 13 tertiary care university hospitals in France  **Period**: September 2016, and November 2016  **Criteria**: Women with a singleton pregnancy who were diagnosed as having gestational diabetes between 24 and 34 weeks of gestation were eligible. | RCT | Glyburide (460), insulin (454) | IADPSG  75-g oral glucose tolerance test resulted in 1 or more abnormal blood glucose values: greater than 92 mg/dL (5.1 mmol/L), greater than 180 mg/dL (10 mmol/L), or greater than 153 mg/dL (8.5 mmol/L) for fasting, 1-hour postprandial, or 2-hour postprandial blood glucose | 10 days dietary intervention | FBG＜95 mg/dL; 2-hour postprandial: ＜120 mg/dL |
| Silva 2012(Silva et al., 2012) | Brazil | **Setting**: Darcy Vargas Maternity Hospital  **Period**: 1 July 2008 to 30 September 2010  **Criteria**: minimum age 18 years, gestational age 11 – 33 weeks, single gestation, fetal abdominal circumference within normal percentile (> 10 % and < 75 %) and absence of other pathologies that might interfere with perinatal results or hypoglycemic therapy | open-label RCT | Metformin (104), glyburide (96) | Not IADPSG (ADA 2009)  ＞95 mg/dL (5.3 mmol/L), ＞180 mg/dL (10.0 mmol/L), and ＞155 mg/dL (8.6 mmol/L) | Diet and physical exercise | FBG＜90 mg/dL and postprandial 120 mg/dL. |
| Simeonova-Krstevska 2018(Simeonova-Krstevska et al., 2018) | Macedonia | **Setting:** Outpatient Department of University Clinic of Endocrinology Diabetes and Metabolic Disorders  **Period:** -  **Criteria:** singleton pregnancies and gave informed consent | RCT | Metformin (48), insulin (101) | IADPSG  75 g OGTT (normal values: a fasting level < 5.1, 1-hour level < 10.0 and 2-hour level < 8.5 mmol/L. | Monitor glucose level | FBG 3.8-5.0 mmol/l and one-hour postprandial blood glucose concentration < 7.8 mmol/l |
| Tempe 2013(Tempe and Mayanglambam, 2013) | India | **Setting**: Maulana Azad Medical College and Lok Nayak Hospital  **Period**: December 2008 to December 2009  **Criteria**: gestational diabetes not responding to diet control; singleton pregnancy; patients with normal liver and kidney function tests; and patients with regular antenatal clinic visits | Prospective comparative RCT | Insulin (32), glyburide (32) | Carpenter and Coustan criteria  A 50-gram oral glucose screening (O’Sullivan test) was followed by a 100-gram oral glucose tolerance test (OGTT) using the National Diabetes Data Group criteria. Isolated fasting glycemia at 100 mg/dL (5.6 mmol/L) was also considered as GDM. | Diabetes diet 25–35 kcal/kg | FBG ≤95 mg/dL and 2-h postprandial plasma glucose≤120 mg/dL |

**Supplementary table 7.** Bias assessment (Master)(Stone et al., 2021)

|  | Format recruitment | | | | Equal retention | | | | | Equal ascertainment | | | | | | | Equal implementation | | | | | | Equal prognosis | | | | | | Sufficient analysis | | | Format recruitment | | | | |
| --- | --- | --- | --- | --- | --- | --- | --- | --- | --- | --- | --- | --- | --- | --- | --- | --- | --- | --- | --- | --- | --- | --- | --- | --- | --- | --- | --- | --- | --- | --- | --- | --- | --- | --- | --- | --- |
|  | 1 | 2 | 3 | 4 | 5 | 6 | 7 | 8 | 9 | 10 | 11 | 12 | 13 | 14 | 15 | 16 | 17 | 18 | 19 | 20 | 21 | 22 | 23 | 24 | 25 | 26 | 27 | 28 | 29 | 30 | 31 | 32 | 33 | 34 | 35 | 36 |
| Ainuddin 2015 |  | √ |  | √ |  |  | √ |  |  | √ | √ | √ |  |  |  |  | √ | √ | √ |  | √ | √ |  |  | √ | √ |  | √ | √ | √ | √ | √ | √ | √ | √ | √ |
| Anjalakshi 2007 | √ | √ |  | √ | √ | √ |  | √ | √ | √ | √ | √ |  |  |  |  |  | √ | √ | √ | √ | √ | √ |  | √ | √ |  |  | √ | √ | √ | √ | √ | √ | √ | √ |
| Arshad 2017 |  | √ | √ | √ |  |  |  |  |  | √ | √ | √ |  |  |  |  | √ | √ | √ |  | √ | √ | √ |  |  |  |  |  | √ | √ | √ | √ | √ | √ | √ | √ |
| Ashoush 2016 |  | √ |  | √ | √ | √ | √ | √ |  | √ | √ | √ |  |  |  |  | √ | √ | √ | √ | √ | √ | √ |  | √ |  |  | √ | √ | √ | √ | √ | √ | √ | √ | √ |
| Behrashi 2016 | √ | √ | √ | √ | √ | √ | √ | √ | √ | √ | √ | √ |  |  |  |  | √ | √ | √ |  | √ | √ | √ |  | √ | √ |  | √ | √ | √ | √ | √ | √ | √ | √ | √ |
| Bertini 2005 | √ | √ | √ | √ | √ | √ | √ | √ | √ | √ | √ | √ |  |  |  |  |  |  | √ |  | √ | √ | √ |  | √ |  |  |  | √ | √ | √ | √ | √ | √ | √ | √ |
| Feghail 2018 | √ | √ | √ | √ | √ | √ | √ | √ | √ | √ | √ |  |  |  |  |  | √ | √ | √ |  | √ | √ | √ |  | √ |  |  |  | √ | √ | √ | √ | √ | √ |  | √ |
| Miriam 2015 | √ | √ | √ | √ | √ | √ | √ | √ |  | √ | √ | √ |  |  |  |  | √ |  | √ |  | √ | √ |  |  |  |  |  |  | √ | √ | √ | √ | √ | √ | √ | √ |
| Mohammad 2019 | √ | √ | √ | √ | √ | √ | √ | √ | √ | √ | √ | √ |  |  |  |  | √ | √ | √ |  |  | √ | √ |  | √ | √ |  |  | √ | √ | √ | √ | √ | √ | √ | √ |
| George 2015 | √ | √ | √ | √ | √ | √ | √ | √ | √ | √ | √ | √ |  |  |  |  | √ | √ | √ |  | √ | √ | √ |  | √ | √ | √ |  | √ | √ | √ | √ | √ | √ | √ | √ |
| Ghomian 2018 | √ | √ | √ | √ |  | √ |  |  | √ | √ | √ | √ |  |  |  |  | √ | √ | √ |  | √ | √ | √ |  | √ |  |  | √ | √ | √ |  | √ | √ | √ | √ | √ |
| Hassan 2012 | √ | √ |  | √ |  |  |  |  |  | √ | √ | √ |  |  |  |  | √ | √ | √ |  | √ | √ | √ |  |  |  |  |  | √ | √ |  |  | √ | √ | √ | √ |
| Ija's 2010 | √ | √ | √ | √ | √ | √ | √ | √ | √ | √ | √ | √ |  |  |  |  | √ | √ | √ |  | √ | √ | √ |  | √ | √ | √ |  | √ | √ |  | √ |  | √ | √ | √ |
| Lain 2009 | √ | √ | √ | √ |  |  |  |  |  |  | √ | √ |  |  |  |  | √ | √ | √ |  | √ | √ | √ |  | √ | √ | √ |  | √ | √ | √ | √ | √ | √ | √ | √ |
| Langer 2000 | √ | √ | √ | √ | √ | √ | √ | √ | √ | √ | √ | √ |  |  |  |  | √ | √ | √ |  | √ | √ |  |  | √ | √ |  |  | √ |  | √ | √ | √ | √ | √ | √ |
| Mirzamoradi 2014 |  | √ | √ | √ | √ | √ | √ | √ | √ | √ | √ | √ |  |  |  |  | √ | √ | √ |  | √ | √ | √ |  | √ | √ | √ |  | √ | √ | √ | √ | √ | √ | √ | √ |
| Moore 2010 | √ | √ | √ | √ | √ | √ | √ | √ | √ | √ | √ | √ |  |  |  |  | √ | √ | √ |  | √ | √ | √ |  | √ | √ |  |  | √ | √ | √ | √ |  | √ | √ | √ |
| Nachum 2017 | √ | √ | √ | √ |  | √ |  | √ | √ | √ | √ | √ |  |  |  |  | √ | √ | √ | √ | √ | √ | √ |  | √ | √ |  |  | √ | √ | √ | √ |  | √ | √ | √ |
| Niromamesh 2012 | √ | √ | √ | √ | √ | √ | √ | √ | √ | √ | √ | √ |  |  |  |  | √ | √ | √ |  | √ | √ | √ |  | √ | √ |  | √ | √ | √ | √ | √ |  | √ | √ | √ |
| Picon-cesar 2021 | √ | √ | √ | √ | √ | √ | √ | √ | √ | √ | √ | √ |  |  |  |  | √ | √ | √ |  | √ | √ | √ |  | √ | √ | √ | √ | √ | √ | √ | √ | √ | √ | √ | √ |
| Rowan 2008 | √ | √ | √ | √ | √ | √ | √ | √ | √ | √ | √ | √ |  |  |  |  |  |  | √ |  | √ | √ | √ |  | √ | √ | √ | √ | √ | √ | √ | √ | √ | √ | √ | √ |
| Ruholamin 2014 | √ | √ | √ | √ | √ | √ | √ | √ | √ | √ | √ | √ |  |  |  |  | √ | √ | √ |  | √ | √ | √ |  | √ |  |  |  |  | √ | √ | √ | √ |  | √ | √ |
| Saleh 2014 | √ | √ | √ | √ | √ | √ | √ | √ | √ | √ | √ | √ |  |  |  |  | √ | √ | √ |  | √ | √ | √ |  | √ | √ |  | √ | √ | √ | √ | √ |  | √ | √ | √ |
| Senat 2018 | √ | √ | √ | √ | √ | √ | √ | √ | √ | √ | √ | √ |  |  |  |  | √ | √ | √ |  | √ | √ | √ |  | √ | √ | √ | √ | √ | √ | √ | √ |  | √ | √ | √ |
| Silva 2012 | √ | √ | √ | √ | √ | √ |  | √ |  | √ | √ | √ |  |  |  |  | √ | √ | √ |  | √ | √ |  |  |  | √ |  | √ | √ | √ | √ | √ | √ | √ | √ | √ |
| Simeonova-Krstevska 2018 |  | √ |  | √ | √ | √ |  |  |  | √ | √ |  |  |  |  |  | √ | √ | √ |  | √ | √ |  |  |  |  |  |  | √ | √ | √ |  |  | √ | √ | √ |
| Spaulonci 2013 | √ | √ | √ | √ | √ | √ | √ | √ | √ | √ | √ | √ |  |  |  |  | √ | √ | √ |  | √ | √ | √ |  | √ | √ | √ | √ | √ | √ | √ | √ | √ | √ | √ | √ |
| Tempe 2013 | √ | √ | √ | √ | √ | √ |  |  |  | √ | √ | √ |  |  |  |  | √ | √ | √ |  | √ | √ | √ |  | √ | √ |  | √ | √ | √ | √ | √ |  | √ | √ | √ |
| Tertti 2013 | √ | √ | √ | √ | √ | √ | √ |  | √ | √ | √ | √ |  |  |  |  | √ | √ | √ |  | √ | √ | √ |  |  | √ | √ | √ | √ | √ | √ | √ | √ | √ | √ | √ |

**Supplementary Table 8.** Maternal outcomes (direct meta-analysis)

| **Outcome** | **Treatment** | **Type** | **Effect size** | **95% CI** |
| --- | --- | --- | --- | --- |
| **Total weight gain (kg)** |  |  |  |  |
| N=9 (2033), I^2^=88% | Metformin-insulin | WMD | -1.53* | -1.69 to -1.37 |
| N=1 (104) | Metformin-glyburide | WMD | -0.30* | -2.92 to -2.32 |
| N=2 (455), I^2^=97% | Insulin-glyburide | WMD | 6.89* | 5.18 to 8.60 |
| **Maternal** **hypoglycemia (<3.3mmol/L)** |  |  |  |  |
| N=2 (295), I^2^=0% | Metformin-insulin | OR | 0.34* | 0.14-0.84 |
| N=2 (195), I^2^=11% | Metformin-glyburide | OR | 0.67 | 0.20-2.31 |
| N=1 (809) | Insulin-glyburide | OR | 0.09* | 0.05-0.16 |
| **Mean fasting plasma glucose (mmol/L)** |  |  |  |  |
| N=11 (2234), I^2^=84% | Metformin-insulin | WMD | -0.06 | -0.15 to 0.03 |
| N=3 (412), I^2^=0% | Metformin-glyburide | WMD | 0.14* | 0.01 to 0.27 |
| N=5 (1354), I^2^=89% | Insulin-glyburide | WMD | 0.14 | -0.10 to 0.37 |
| **M****ean postprandial (mmol/L)** |  |  |  |  |
| N=10 (2184), I^2^=100% | Metformin-insulin | WMD | -0.00 | -0.64 to 0.64 |
| N=3 (412), I^2^=0% | Metformin-glyburide | WMD | -0.08 | -0.26 to 0.10 |
| N=6 (1377), I^2^=61% | Insulin-glyburide | WMD | 0.14* | 0.03 to 0.24 |
| **G****lycemic target unmet (%)** |  |  |  |  |
| N=3 (617), I^2^=95% | Metformin-insulin | OR | 3.18* | 2.15-4.69 |
| N=1 (46) | Metformin-glyburide | OR | 0.27 | 0.05-1.51 |
| N=0 | Insulin-glyburide | OR | - | - |

**Supplementary Table 9.** Feta outcomes (direct meta-analysis)

| **Outcome** | **Treatment** | **Type** | **Effect size** | **95% CI** |
| --- | --- | --- | --- | --- |
| **Birth weight (kg)** |  |  |  |  |
| N=14 (2645), I^2^=86% | Metformin-insulin | WMD | -113.47* | -203.59 to -23.36 |
| N=3 (412), I^2^=78% | Metformin-glyburide | WMD | -46.12 | -209.84 to 117.61 |
| N=6 (1892), I^2^=30% | Insulin-glyburide | WMD | -40.35 | -89.348 to 8.64 |
| **LGA (%)** |  |  |  |  |
| N=9 (2001), I^2^=22% | Metformin-insulin | OR | 0.74* | 0.56-0.93 |
| N=1 (104) | Metformin-glyburide | OR | 1.60 | 0.56-4.60 |
| N=5 (1124), I^2^=61% | Insulin-glyburide | OR | 0.66 | 0.29-1.49 |
| **Neonatal death (%)** |  |  |  |  |
| N=8 (1702), I^2^=0% | Metformin-insulin | OR | 0.38 | 0.07-2.01 |
| N=1 (159) | Metformin-glyburide | OR | No events | No events |
| N=1 (404), | Insulin-glyburide | OR | 0.99 | 0.06-15.94 |
| **Still birth (%)** |  |  |  |  |
| N=2 | Metformin-insulin | OR | - | - |
| N=0 | Metformin-glyburide | OR | - | - |
| N=1 (404) | Insulin-glyburide | OR | 0.99 | 0.06-15.94 |
| **NICU admission (%)** |  |  |  |  |
| N=12 (2483), I^2^=0% | Metformin-insulin | OR | 0.82 | 0.67-1.02 |
| N=1 (151) | Metformin-glyburide | OR | 4.11 | 0.45-37.70 |
| N=6 (404), I^2^=0% | Insulin-glyburide | OR | 1.13 | 0.78 -1.64 |
| **H****ypoglycemia within 1h of birth (＜2.22mmol/L, %)** |  |  |  |  |
| N=14 (2726), I^2^=0% | Metformin-insulin | OR | 0.66* | 0.53-0.81 |
| N=4 (458), I^2^=76% | Metformin-glyburide | OR | 0.94 | 0.04-23.61 |
| N=6 (2100), I^2^=39% | Insulin-glyburide | OR | 0.61* | 0.44-0.84 |

**Supplementary Table 10.** Pregnancy outcomes (direct meta-analysis)

| **Outcome** | **Treatment** | **Type** | **Effect size** | **95% CI** |
| --- | --- | --- | --- | --- |
| **Assisted labor (non-cesarean) %** |  |  |  |  |
| N=7 (956), I^2^=0% | Metformin-insulin | OR | 1.22 | 0.77-1.94 |
| N=0 | Metformin-glyburide | OR | - | - |
| N=1 (809) | Insulin-glyburide | OR | 0.86 | 0.59-1.26 |
| **Cesarean section (%)** |  |  |  |  |
| N=12 (1828), I^2^= 43% | Metformin-insulin | OR | 0.73* | 0.60-0.89 |
| N=4 (458), I^2^=32% | Metformin-glyburide | OR | 1.54 | 1.00-2.38 |
| N=3 (1425), I^2^=0% | Insulin-glyburide | OR | 0.86 | 0.59-1.26 |
| **Pre-term delivery (%)** |  |  |  |  |
| N=5 (768), I^2^=0% | Metformin-insulin | OR | 0.58 | 0.31-1.08 |
| N=2 (263), I^2^=0% | Metformin-glyburide | OR | 1.95 | 0.63-6.09 |
| N=1 (809) | Insulin-glyburide | OR | 0.58 | 0.31-1.08 |
| **Pregnancy induced hypertension (%)** |  |  |  |  |
| N=6 (1525), I^2^=5% | Metformin-insulin | OR | 0.63 | 0.40-1.00 |
| N=2 (263), I^2^=0% | Metformin-glyburide | OR | 0.63 | 0.26-1.51 |
| N=1 (523) | Insulin-glyburide | OR | 1.47 | 0.84-2.57 |
| **Pre-eclampsia (%)** |  |  |  |  |
| N=6 (1514), I^2^=0% | Metformin-insulin | OR | 0.76 | 0.52-1.13 |
| N=1 (149) | Metformin-glyburide | OR | 0.65 | 0.11-4.00 |
| N=0 | Insulin-glyburide | OR |  |  |
| **Emergency cesarean section (%)** |  |  |  |  |
| N=2 (297), I^2^=39% | Metformin-insulin | OR | 1.33 | 0.78-2.27 |
| N=0 | Metformin-glyburide | OR | - | - |
| N=1 (809) | Insulin-glyburide | OR | 0.73 | 0.49-1.07 |

## Supplementary Figures


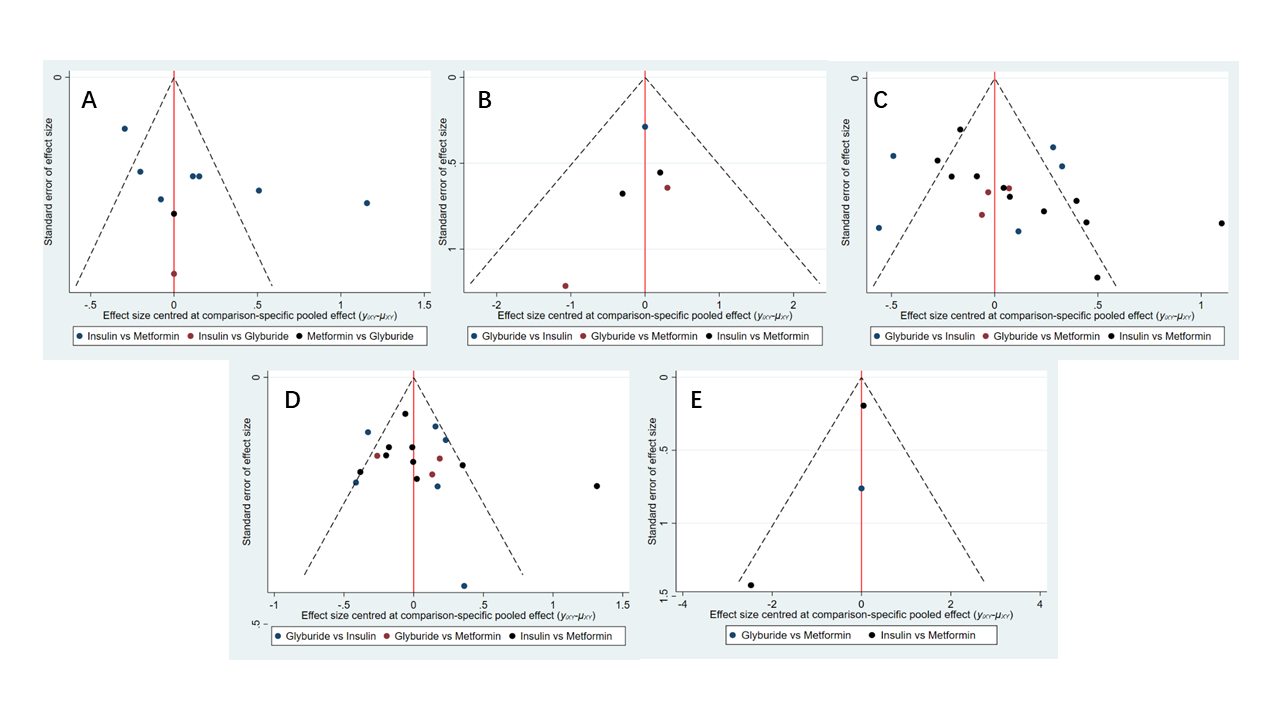


**Supplementary Figure 1.** Funnel plots of maternal metabolic outcomes. (A) is the funnel plots of total GWG, (B) for maternal hypoglycaemia, (C) for mean plasma fasting glucose, (D) for the mean postprandial glucose, (E) for the glycaemic targets unmet.


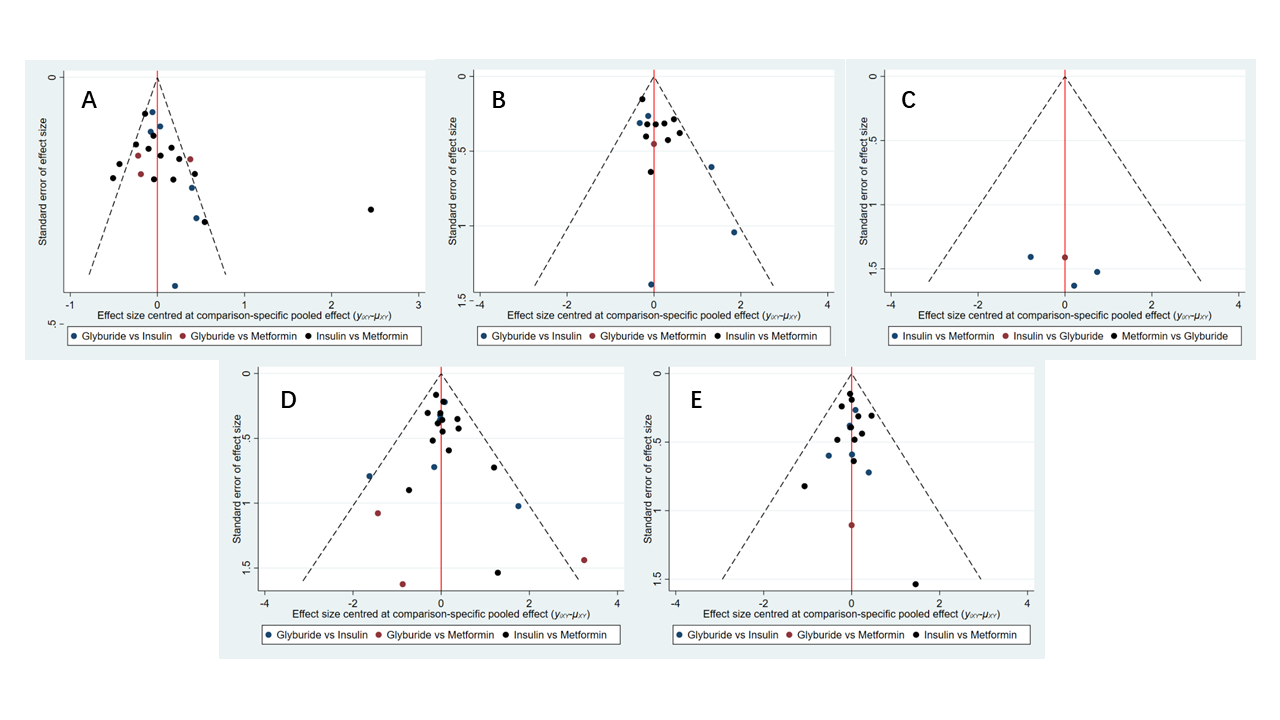


**Supplementary Figure 2.** Funnel plots of fetal outcomes. (A) is the funnel plots of birth weight (kg), (B) for maternal large for gestational age, (C) for neonatal death, (D) for neonatal hypoglycaemia, (E) for NICU admission.


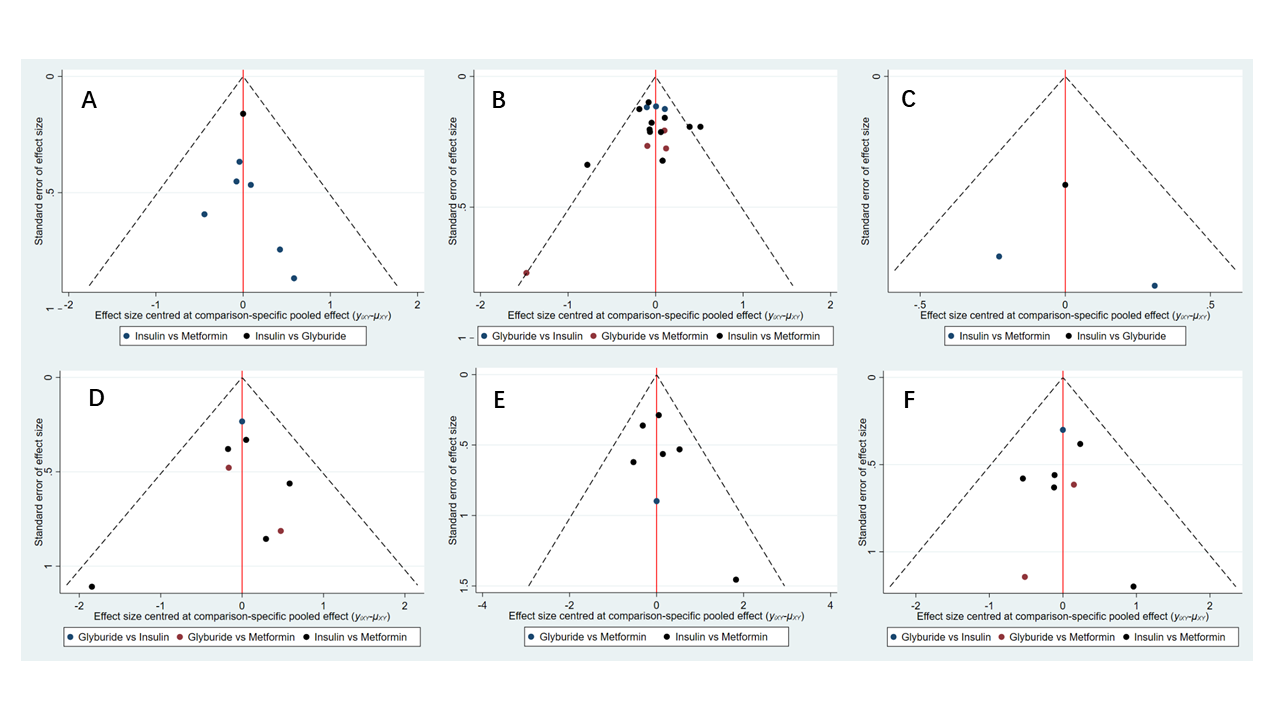


**Supplementary Figure 3.** Funnel plots of pregnancy outcomes. (A) is the funnel plots of assisted labour, (B) for caesarean section, (C) for emergency c-section, (D) for pregnancy induced hypertension (PIH), (E) for pre-eclampsia, (F) for preterm delivery.


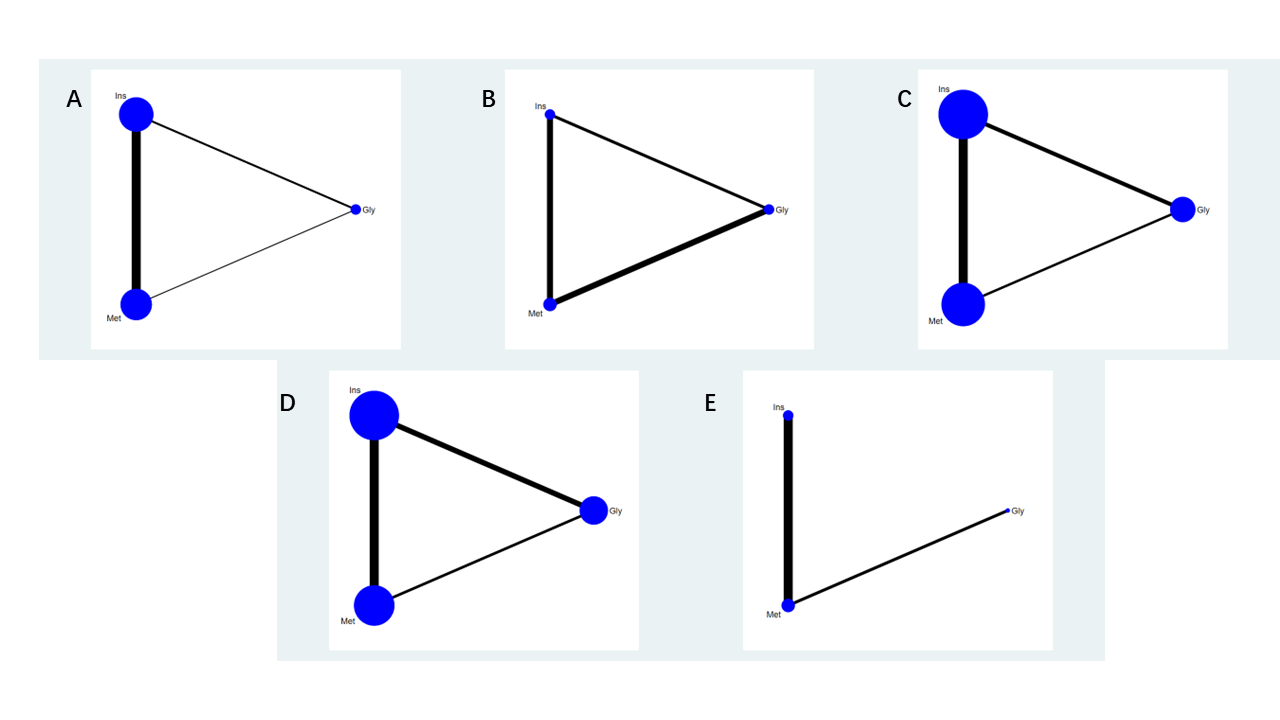


**Supplementary Figure 4.** Network maps of maternal metabolic outcomes. (A) is the network map of total GWG, (B) for maternal hypoglycaemia, (C) for mean plasma fasting glucose, (D) for the mean postprandial glucose, (E) for the glycaemic targets unmet.


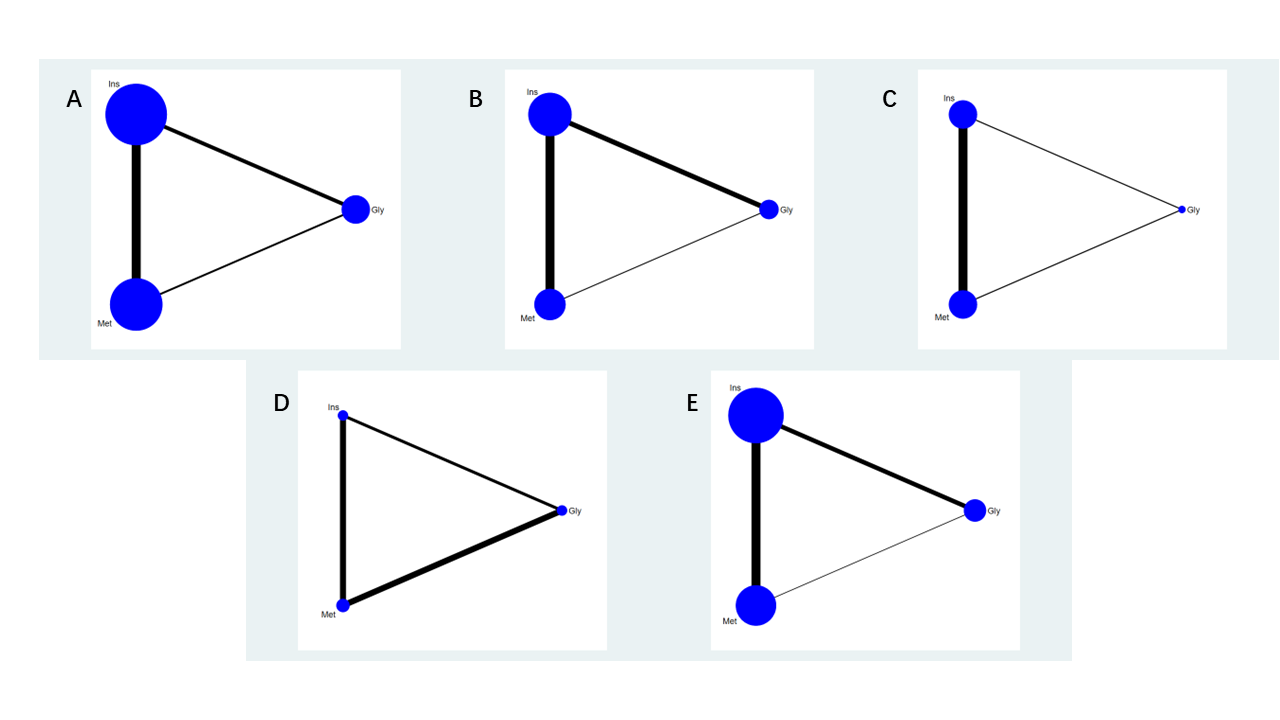


**Supplementary Figure 5.** Network maps of fetal outcomes. (A) is the network map of birth weight (kg), (B) for maternal large for gestational age, (C) for neonatal death, (D) for neonatal hypoglycaemia, (E) for NICU admission.


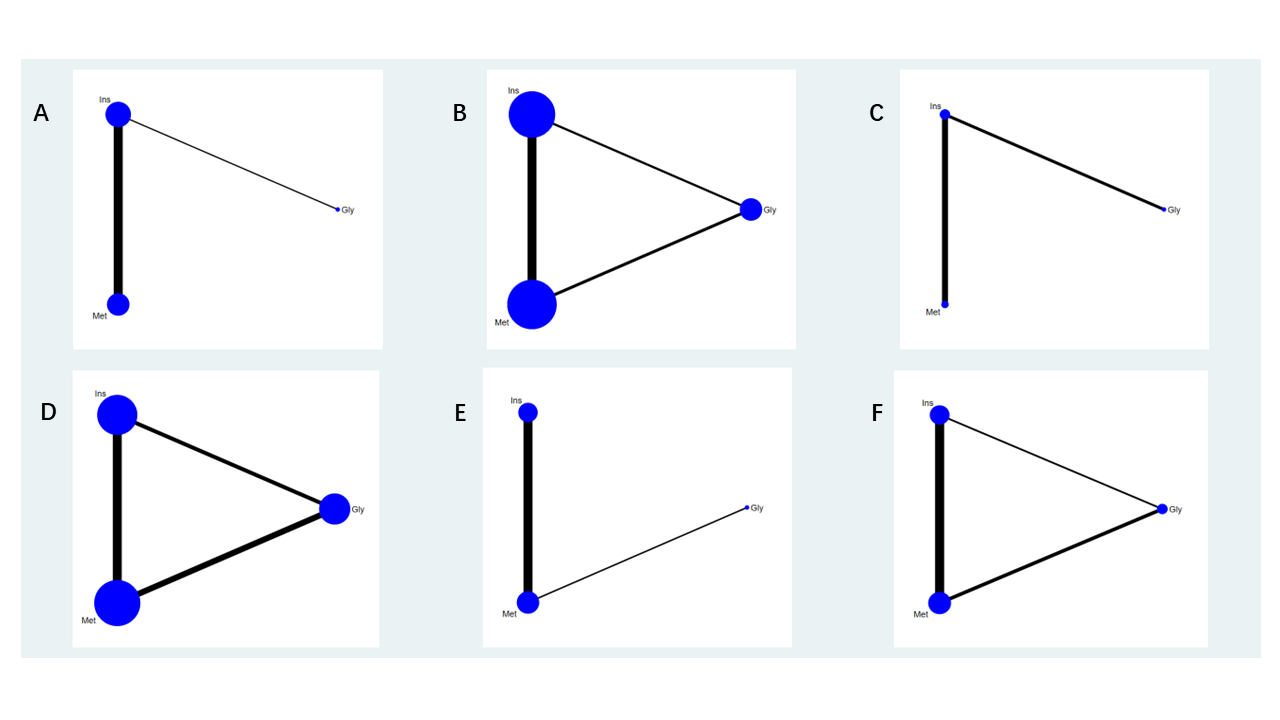


**Supplementary Figure 6.** Network maps of pregnancy outcomes. (A) is the network map of assisted labour, (B) for caesarean section, (C) for emergency c-section, (D) for pregnancy induced hypertension (PIH), (E) for pre-eclampsia, (F) for preterm delivery.

AINUDDIN, J., KARIM, N., HASAN, A. A. & NAQVI, S. A. 2015. Metformin versus insulin treatment in gestational diabetes in pregnancy in a developing country: a randomized control trial. *Diabetes Res Clin Pract,* 107**,** 290-9.

ANJALAKSHI, C., BALAJI, V., BALAJI, M. S. & SESHIAH, V. 2007. A prospective study comparing insulin and glibenclamide in gestational diabetes mellitus in Asian Indian women. *Diabetes Res Clin Pract,* 76**,** 474-5.

ARSHAD, R., KHANAM, S., SHAIKH, F. & KARIM, N. 2017. Feto-maternal outcomes and Glycemic control in Metformin versus insulin treated Gestational Diabetics. *Pak J Med Sci,* 33**,** 1182-1187.

ASHOUSH, S., EL-SAID, M., FATHI, H. & ABDELNABY, M. 2016. 1.73分 Identification of metformin poor responders, requiring supplemental insulin, during randomization of metformin versus insulin for the control of gestational diabetes mellitus. *J Obstet Gynaecol Res,* 42**,** 640-7.

BEHRASHI, M., SAMIMI, M., GHASEMI, T., SABERI, F. & ATOOF, F. 2016. Comparison of Glibenclamide and Insulin on Neonatal Outcomes in Pregnant Women with Gestational Diabetes. *Int J Prev Med,* 7**,** 88.

BERTINI, A. M., SILVA, J. C., TABORDA, W., BECKER, F., LEMOS BEBBER, F. R., ZUCCO VIESI, J. M., AQUIM, G. & ENGEL RIBEIRO, T. 2005. Perinatal outcomes and the use of oral hypoglycemic agents. *J Perinat Med,* 33**,** 519-23.

FEGHALI, M., ATLASS, J., ABEBE, K. Z., COMER, D., CATOV, J., CARITIS, S., ARSLANIAN, S. & SCIFRES, C. 2021. Treatment of Gestational Diabetes Mellitus and Offspring Early Childhood Growth. *J Clin Endocrinol Metab,* 106**,** e1849-e1858.

GEORGE, A., MATHEWS, J. E., SAM, D., BECK, M., BENJAMIN, S. J., ABRAHAM, A., ANTONISAMY, B., JANA, A. K. & THOMAS, N. 2015. Comparison of neonatal outcomes in women with gestational diabetes with moderate hyperglycaemia on metformin or glibenclamide--a randomised controlled trial. *Aust N Z J Obstet Gynaecol,* 55**,** 47-52.

GHOMIAN, N., VAHED, S. H. M., FIROUZ, S., YAGHOUBI, M. A., MOHEBBI, M. & SAHEBKAR, A. 2019. The efficacy of metformin compared with insulin in regulating blood glucose levels during gestational diabetes mellitus: A randomized clinical trial. *J Cell Physiol,* 234**,** 4695-4701.

HASSAN, J. A., KARIM, N. & SHEIKH, Z. 2012. Metformin prevents macrosomia and neonatal morbidity in gestational diabetes. *Pakistan Journal of Medical Sciences,* 28**,** 384-389.

IJäS, H., VääRäSMäKI, M., MORIN-PAPUNEN, L., KERAVUO, R., EBELING, T., SAARELA, T. & RAUDASKOSKI, T. 2011. Metformin should be considered in the treatment of gestational diabetes: a prospective randomised study. *Bjog,* 118**,** 880-5.

JIANG, Y. F., CHEN, X. Y., DING, T., WANG, X. F., ZHU, Z. N. & SU, S. W. 2015. Comparative efficacy and safety of OADs in management of GDM: Network meta-analysis of randomized controlled trials. *Journal of Clinical Endocrinology and Metabolism,* 100**,** 2071-2080.

LAIN, K. Y., GARABEDIAN, M. J., DAFTARY, A. & JEYABALAN, A. 2009. Neonatal adiposity following maternal treatment of gestational diabetes with glyburide compared with insulin. *Am J Obstet Gynecol,* 200**,** 501.e1-6.

LANGER, O., CONWAY, D. L., BERKUS, M. D., XENAKIS, E. M. & GONZALES, O. 2000. A comparison of glyburide and insulin in women with gestational diabetes mellitus. *N Engl J Med,* 343**,** 1134-8.

MIRIAM GEORGE FENN, M. I., MARY GEORGE, SARA KORULA 2015. *COMPARISON OF METFORMIN WITH GLYBURIDE IN GESTATIONAL DIABETES: A DOUBLE BLIND RANDOMISED CLINICAL TRIAL,* J of Evolution of Med and Dent Sci/.

MIRZAMORADI, M., HEIDAR, Z., FAALPOOR, Z., NAEIJI, Z. & JAMALI, R. 2015. Comparison of glyburide and insulin in women with gestational diabetes mellitus and associated perinatal outcome: a randomized clinical trial. *Acta Med Iran,* 53**,** 97-103.

MOHAMMAD GALAL, W. M. E. B., LOBNA SHERIF 2019. *Metformin Versus Insulin in Treatment of Gestational Diabetes Mellitus: A Randomized Controlled Trial*.

MOORE, L. E., CLOKEY, D., RAPPAPORT, V. J. & CURET, L. B. 2010. 7.66分 Metformin compared with glyburide in gestational diabetes: a randomized controlled trial. *Obstet Gynecol,* 115**,** 55-59.

MUSA, O. A. H., SYED, A., MOHAMED, A. M., CHIVESE, T., CLARK, J., FURUYA-KANAMORI, L., XU, C., TOFT, E., BASHIR, M., ABOU-SAMRA, A. B., THALIB, L. & DOI, S. A. 2021. Metformin is comparable to insulin for pharmacotherapy in gestational diabetes mellitus: A network meta-analysis evaluating 6046 women. *Pharmacol Res,* 167**,** 105546.

NACHUM, Z., ZAFRAN, N., SALIM, R., HISSIN, N., HASANEIN, J., GAM ZE LETOVA, Y., SULEIMAN, A. & YEFET, E. 2017. Glyburide Versus Metformin and Their Combination for the Treatment of Gestational Diabetes Mellitus: A Randomized Controlled Study. *Diabetes Care,* 40**,** 332-337.

NIROMANESH, S., ALAVI, A., SHARBAF, F. R., AMJADI, N., MOOSAVI, S. & AKBARI, S. 2012. Metformin compared with insulin in the management of gestational diabetes mellitus: a randomized clinical trial. *Diabetes Res Clin Pract,* 98**,** 422-9.

PICóN-CéSAR, M. J., MOLINA-VEGA, M., SUáREZ-ARANA, M., GONZáLEZ-MESA, E., SOLA-MOYANO, A. P., ROLDAN-LóPEZ, R., ROMERO-NARBONA, F., OLVEIRA, G., TINAHONES, F. J. & GONZáLEZ-ROMERO, S. 2021. Metformin for gestational diabetes study: metformin vs insulin in gestational diabetes: glycemic control and obstetrical and perinatal outcomes: randomized prospective trial. *Am J Obstet Gynecol,* 225**,** 517.e1-517.e17.

ROWAN, J. A., HAGUE, W. M., GAO, W., BATTIN, M. R., MOORE, M. P. & MI, G. T. I. 2008. Metformin versus insulin for the treatment of gestational diabetes. *N Engl J Med,* 358**,** 2003-15.

RUHOLAMIN, S., ESHAGHIAN, S. & ALLAME, Z. 2014. Neonatal outcomes in women with gestational diabetes mellitus treated with metformin in compare with insulin: A randomized clinical trial. *J Res Med Sci,* 19**,** 970-5.

SALEH, H. S., ABDELSALAM, W. A., MOWAFY, H. E. & ABD ELHAMEID, A. A. 2016. Could Metformin Manage Gestational Diabetes Mellitus instead of Insulin? *Int J Reprod Med,* 2016**,** 3480629.

SéNAT, M. V., AFFRES, H., LETOURNEAU, A., COUSTOLS-VALAT, M., CAZAUBIEL, M., LEGARDEUR, H., JACQUIER, J. F., BOURCIGAUX, N., SIMON, E., ROD, A., HéRON, I., CASTERA, V., SENTILHES, L., BRETELLE, F., ROLLAND, C., MORIN, M., DERUELLE, P., DE CARNE, C., MAILLOT, F., BEUCHER, G., VERSPYCK, E., DESBRIERE, R., LABOUREAU, S., MITANCHEZ, D. & BOUYER, J. 2018. 高分Effect of Glyburide vs Subcutaneous Insulin on Perinatal Complications Among Women With Gestational Diabetes: A Randomized Clinical Trial. *Jama,* 319**,** 1773-1780.

SILVA, J. C., FACHIN, D. R., CORAL, M. L. & BERTINI, A. M. 2012. Perinatal impact of the use of metformin and glyburide for the treatment of gestational diabetes mellitus. *J Perinat Med,* 40**,** 225-8.

SIMEONOVA-KRSTEVSKA, S., BOGOEV, M., BOGOEVA, K., ZISOVSKA, E., SAMARDZISKI, I., VELKOSKA-NAKOVA, V., LIVRINOVA, V., TODOROVSKA, I., SIMA, A. & BLAZEVSKA-SILJANOSKA, V. 2018. Maternal and Neonatal Outcomes in Pregnant Women with Gestational Diabetes Mellitus Treated with Diet, Metformin or Insulin. *Open Access Maced J Med Sci,* 6**,** 803-807.

SPAULONCI, C. P., BERNARDES, L. S., TRINDADE, T. C., ZUGAIB, M. & FRANCISCO, R. P. 2013. Randomized trial of metformin vs insulin in the management of gestational diabetes. *Am J Obstet Gynecol,* 209**,** 34 e1-7.

STONE, J. C., GLASS, K., CLARK, J., RITSKES-HOITINGA, M., MUNN, Z., TUGWELL, P. & DOI, S. A. R. 2021. The MethodologicAl STandards for Epidemiological Research (MASTER) scale demonstrated a unified framework for bias assessment. *J Clin Epidemiol,* 134**,** 52-64.

TEMPE, A. & MAYANGLAMBAM, R. D. 2013. Glyburide as treatment option for gestational diabetes mellitus. *J Obstet Gynaecol Res,* 39**,** 1147-52.

TERTTI, K., EKBLAD, U., KOSKINEN, P., VAHLBERG, T. & RöNNEMAA, T. 2013. Metformin vs. insulin in gestational diabetes. A randomized study characterizing metformin patients needing additional insulin. *Diabetes Obes Metab,* 15**,** 246-51.

YU, D. Q., XU, G. X., TENG, X. Y., XU, J. W., TANG, L. F., FENG, C., RAO, J. P., JIN, M. & WANG, L. Q. 2021. Glycemic control and neonatal outcomes in women with gestational diabetes mellitus treated using glyburide, metformin, or insulin: a pairwise and network meta-analysis. *BMC Endocrine Disorders,* 21.
